# A review of current effective COVID-19 testing methods and quality control

**DOI:** 10.1007/s00203-023-03579-9

**Published:** 2023-05-17

**Authors:** Lijia Cheng, Liang Lan, Murugan Ramalingam, Jianrong He, Yimin Yang, Min Gao, Zheng Shi

**Affiliations:** Clinical Medical College & Affiliated Hospital, School of Basic Medical Sciences, Chengdu University, Chengdu, 610106 China

**Keywords:** COVID-19, SARS-CoV-2, Point-of-care testing, Quality control

## Abstract

COVID-19 is a highly infectious disease caused by the SARS-CoV-2 virus, which primarily affects the respiratory system and can lead to severe illness. The virus is extremely contagious, early and accurate diagnosis of SARS-CoV-2 is crucial to contain its spread, to provide prompt treatment, and to prevent complications. Currently, the reverse transcriptase polymerase chain reaction (RT-PCR) is considered to be the gold standard for detecting COVID-19 in its early stages. In addition, loop-mediated isothermal amplification (LMAP), clustering rule interval short palindromic repeats (CRISPR), colloidal gold immunochromatographic assay (GICA), computed tomography (CT), and electrochemical sensors are also common tests. However, these different methods vary greatly in terms of their detection efficiency, specificity, accuracy, sensitivity, cost, and throughput. Besides, most of the current detection methods are conducted in central hospitals and laboratories, which is a great challenge for remote and underdeveloped areas. Therefore, it is essential to review the advantages and disadvantages of different COVID-19 detection methods, as well as the technology that can enhance detection efficiency and improve detection quality in greater details.

## Introduction

Seven different types of human coronaviruses have been discovered to cause infectious diseases. Among them, HCOV-299E, HCOV-NL63, HCOV-OC43 and HCOV-HKU1 can cause mild disease, while SARS-COV, MERS-COV, and SARS-CoV-2 have severe symptoms and resemble the solar corona (Malik 2020). SARS-CoV-2, a member of the coronavirus clade, has an RNA genome size of 29.9 KB. Phylogenetic studies have shown that SARS-COV and SARS-CoV-2 shared a common ancestor and belonged to the same branch. SARS-CoV-2 was discovered by Chinese researchers to have a bat origin, and it shares an 88% nucleotide sequence homology with two other coronaviruses that are similar to SARS (BAT-SL-CoVZC45 and BAT-SL-CovZXC2) (Lu and Zhao 2020). SARS-CoV-2 was first reported on December 31, 2019, in Wuhan, Hubei Province, China. It quickly spread over the world and is now being fought in more than 200 nations as COVID-19 (Wang et al. 2020a, b, c). The International Committee on Viral Classification has identified the virus's causal agent as severe acute respiratory syndrome coronavirus 2, which was formerly known as the 2019 new coronavirus (2019-nCoV) (SARS-CoV-2). On January 30, 2020, the World Health Organization (WHO) declared the COVID-19 outbreak a public health emergency of worldwide significance. The incidence of COVID-19 infections and fatalities was drastically rising, with a cumulative total of 673,519,283 confirmed COVID-19 cases and 6,819,364 cumulative deaths worldwide as of 2 April 2023. COVID-19 is a kind of respiratory disease that is mainly transmitted through contact and inhalation. An uninfected person can become infected by touching a contaminated object before touching their eyes, nose, or mouth. Infection can also occur by inhaling droplets from an infected person who coughs or sneezes, or speak or breathe in close proximity to an infected person. The infection has a range of clinical manifestations, including cough, fever, severe headache, and dyspnea. In severe cases, organ function damage can be caused, such as heart, kidney, or liver failure, acute respiratory distress syndrome, or even death (Huang et al. 2020a, b). COVID-19 has caused great losses to people's lives and the global economy.

**Fig. 1:**
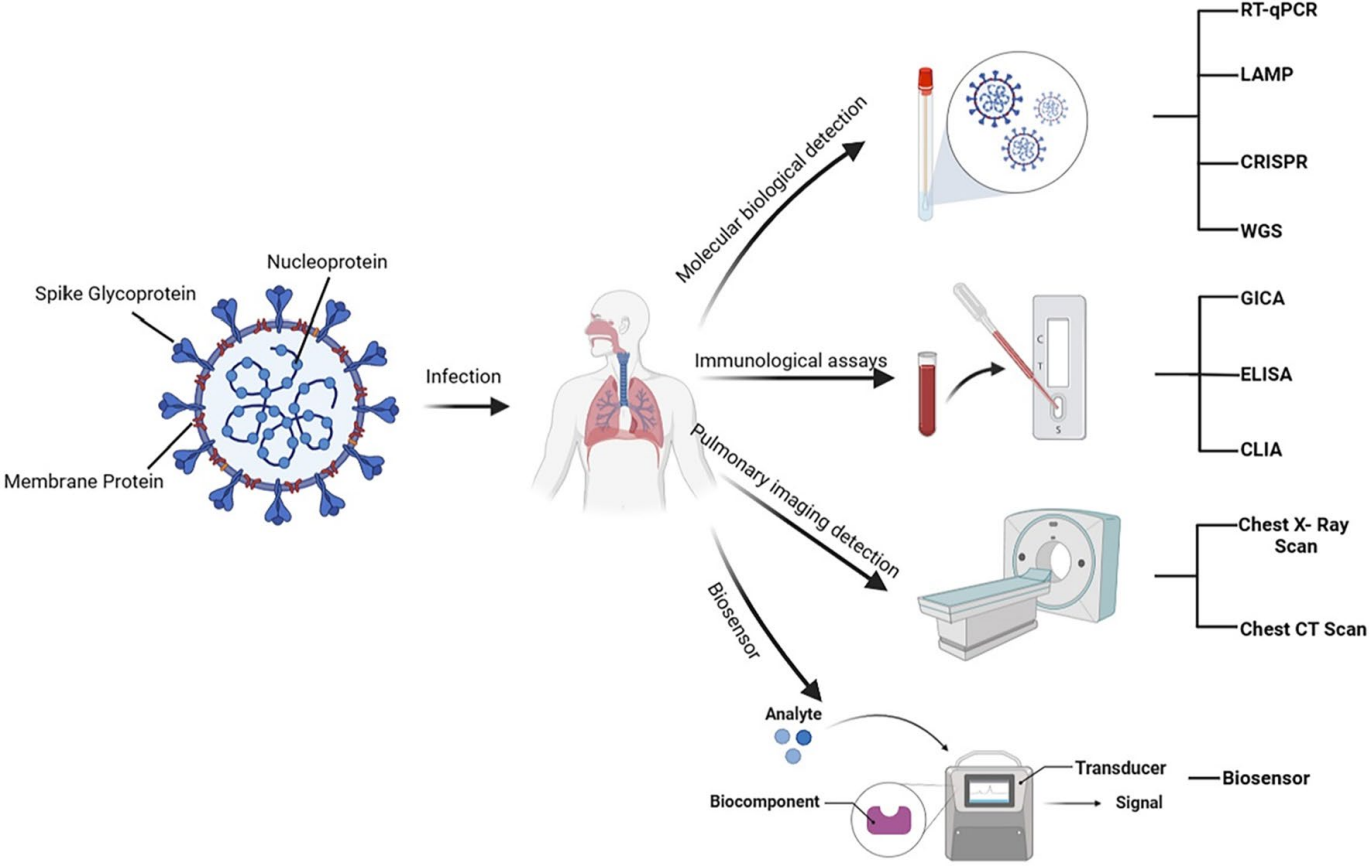
Schematic illustration of strategies for the detection of COVID-19 patients

SARS-CoV-2 consists of four structural proteins: surface glycoprotein (S), envelope protein (E), membrane protein (M), and nucleocapsid protein (N) (Wu et al. 2020a, b). S protein is composed of S1 and S2 subunits. S1 is a major surface antigen that interacts to host cell receptors. S2 mediates membrane fusion between host and SARS-CoV-2 membranes, allowing SARS-CoV-2 RNA genomes to enter host cells. The primary structural protein of SARS-CoV-2 is N protein, which is involved in the replication and transcription of viral RNA, formation and maintenance of ribonucleic protein complexes, and packaging of enveloped genomes into virus particles (Lan et al. 2020). In addition, it can regulate the host cell cycle to promote virus proliferation and transmission (McBride et al. 2014). The E protein is the smallest main structural protein of SARS-CoV-2, which ranges from 8.4 to 12 KDa and participates in the assembly and release of viral particles in host cells. The most prevalent protein in virus particles, M protein, is crucial for viral assembly and internal stability (Lan et al. 2020). Various techniques have been developed to detect the coronavirus. The most used technique for SARS-CoV-2 direct diagnosis among them is nucleic acid amplification by RT-PCR. Three conserved sections of the SARS-CoV-2 genome (E, N and ORF1ab) are selected as reliable targets for all types of PCR analyses. Meanwhile, the most crucial targets for serological immunoassay are S and N proteins. In addition to laboratory-based RT-PCR and immunoassays, various emerging methods such as electrochemical sensors, point-of-care testing, etc. In this paper, we will review the recently published detection methods for SARS-CoV-2 (Fig. 1), introduce the basic principles of each detection method, compares their detection effects, and discuss the limitations of these methods. The cost, trained personnel, time and other details required by different detection methods are summarized in Table 1. In addition, we will also evaluate how to improve test efficiency and test quality, to detect infected patients early and timely, thus preventing the virus's spread and reducing the incidence of complications.

**Table 1:** A comparison among the main detecting methods for COVID-19

Detection methods	Prime apparatus	LOD	Mean sensitivity	Time	The need for trained personnel	Cost	Advantage	Disadvantage	Future prospect	References
Molecular biological Detection	PCR apparatus	10–500 copies/reaction	75%	1–4 h	At least biosafety Level 2 (BSL-2) laboratories, specialized equipment, and trained laboratory personnel are required	High	High sensitivity and high specificity	Complex, expensive, time consuming, false negative, requiring expensive laboratory instrumentation and highly skilled laboratory personnel	RT-PCR will still be the gold standard of detection in the future, and the rapid development of polynucleic acid detection technology will provide rapid, simple and diversified nucleic acid detection schemes	Udugama et al. (2020); Ai et al. (2020a, b); Carter et al. (2020); Urrutia-Cabrera et al. (2021); García-Bernalt Diego et al. (2022)
Immunological assays	ELISA machine, test strips or kits	10–20 copies/reaction	56%	15–20 min	Highly specialized skills, equipment, and BSL-3 facilities are required	5 ~ 10$ per person	Strong specificity, low false positive rate, low cost, convenient operation, fast	The sensitivity varies significantly with different assays, initial diagnosis of infection is limited	Due to high specificity and strong correlation with virus, the immunological testing holds great potential to become a preferred tool to assess thresholds of protective immunity after infection and vaccination	Lambert-Niclot et al. (2020); Lai et al. (2021); Wang et al. (2022)
Pulmonary Imaging Detection	CT machine, supersonic reflectoscope	–	96%	1–2 h	Solid professional knowledge and rich clinical experience	150–3000$ per person	High efficiency and auxiliary diagnosis	Poor specificity, the expensive price, inconvenient, easy to infection	Chest CT can also be used to diagnose acute respiratory diseases (ARDS) and extrapulmonary complications caused by COVID-19. In addition, it will play a greater value in the indication of disease development and evaluation of discharge	Fang et al. (2020); Campagnano et al. (2021); Pourabhari Langroudi et al. (2022)

## Molecular biological detection

**Fig. 2:**
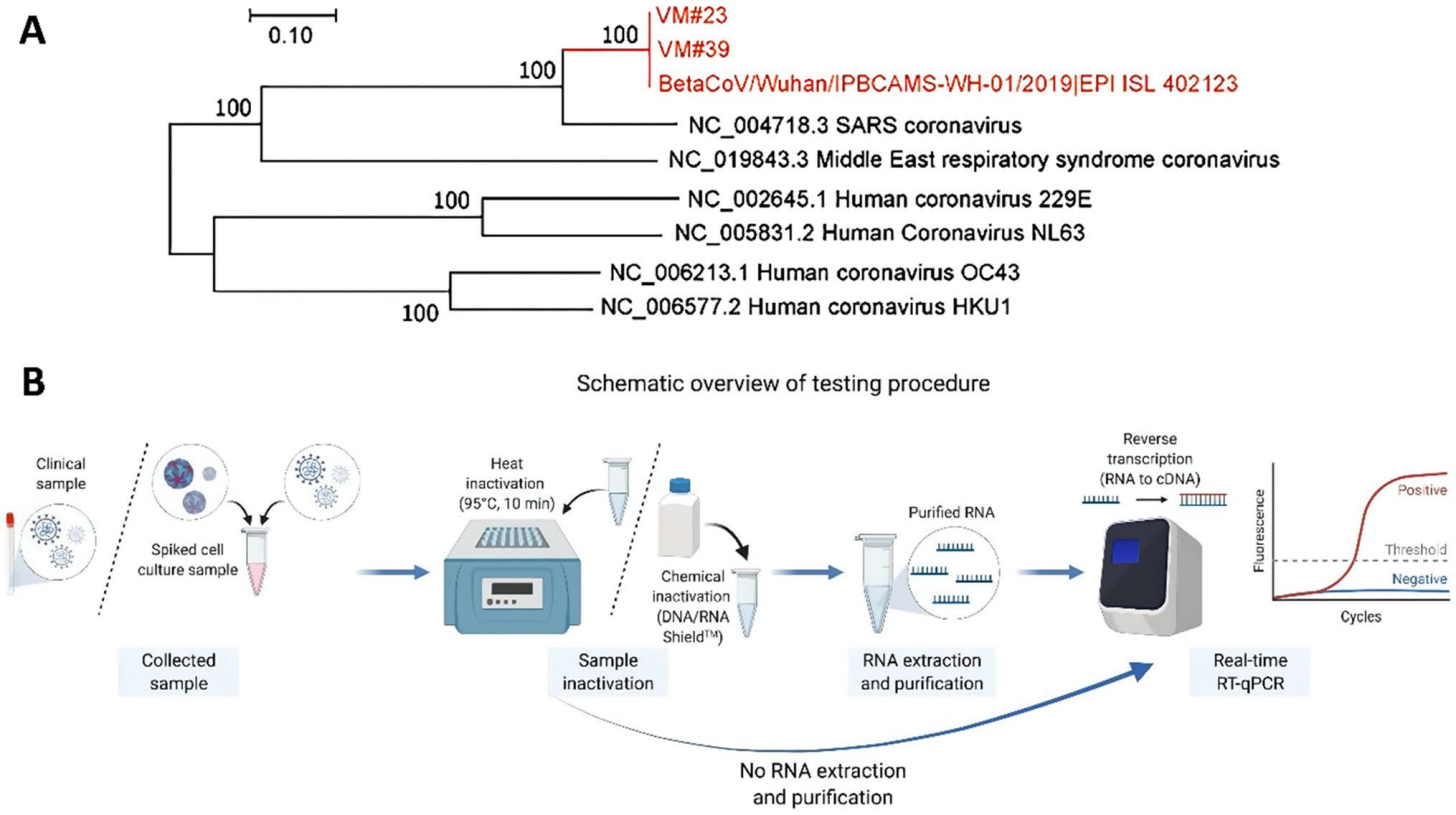
Phylogenetic tree of SARS-CoV-2 s and other pathogenic CoVs and process diagram for real-time RT-qPCR. **A** SARS-CoV-2 phylogenetic tree and other pathogenic CoVs (Hou et al. 2020). **B** Overview schematic of the real-time RT-qPCR workflow (Mao et al. 2021)

In recent years, the highly contagious SARS-CoV-2 has quickly spread worldwide, causing significant harm. Detecting the source of the infection and preventing its spread at an early stage is crucial. Nucleic acid tests for viruses are highly specific and accurate, making them more reliable than many other tests. Currently, the most popular molecular biological tests, such as reverse transcriptase polymerase chain reaction (RT-PCR), loop-mediated isothermal amplification (LAMP), clustered regularly interspaced short palindromic repeats (CRISPR), whole-genome sequencing (WGS), cartridge based nucleic acid amplification test (CBNAAT), gene chips and so on, have been designed for quick COVID-19 assessment and detection (Ai et al. 2020a, b). Based on the complete genome, phylogenetic analysis indicates that the SARS-CoV-2 clusters was more like bat-SARS-CoV, rather than other human coronaviruses (HCoVs) (Fig. 2A). But many of the symptoms of SARS-CoV-2 infection are similar to those of HCoVs. Therefore, it is necessary to pay special attention on the types of coronaviruses. Most molecular detection techniques rely on understanding the viral genome, protein composition, and how they change during and after infection (Udugama et al. 2020). Therefore, combining and optimizing different molecular detection techniques is essential in increasing the clinical detection rate.

### Reverse transcriptase polymerase chain reaction (RT-PCR)

The SARS-CoV-2 virus’s DNA fragments can be amplified by RT-PCR, which involves using reverse transcriptase to produce cDNA from extracted RNA and then amplifying the cDNA using PCR. This method can be used qualitatively and quantitatively, with a significant advantage in detecting SARS-CoV-2. The second-generation PCR technology uses probe-based real-time quantitative PCR, where fluorescent labeled probes detect emitted fluorescence during the PCR reaction, and the initial concentration can be calculated quantitatively from the curve generated by nucleic acid amplification (Fig. 2B). This method is widely applied in many countries (Liu et al. 2020a, b; Loeffelholz and Tang 2020).

**Fig. 3:**
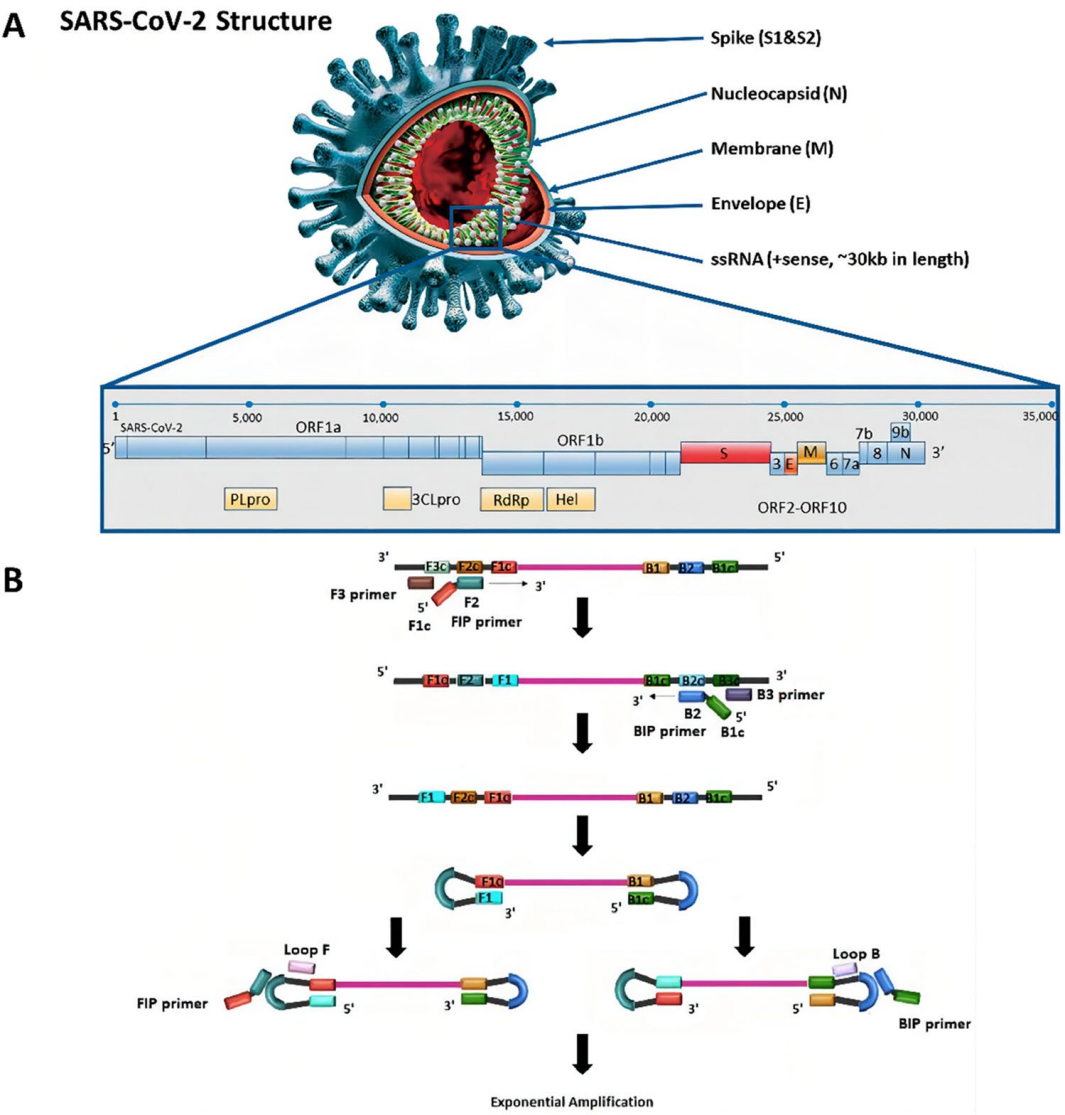
The SARS-CoV-2 structure and major gene sequences and loop-mediated isothermal amplification diagram. **A** Structure and major gene sequences of SARS-CoV-2 (Kubina and Dziedzic 2020). **B** Schematic principle of loop-mediated isothermal amplification (Chaouch 2021)

The SARS-CoV-2 virus’s target gene include nucleocapsid protein (N), envelope protein (E), RdRp and ORF1ab, among others (Fig. 3A). The results of detection depend on the design of the gene primer probes used and the accuracy of RT-PCR detection can be significantly improved by choosing and combining new amplification sites in primer design. In general, WHO recommended the use of RT-PCR targeting the E gene, and validating it with the RdRp gene (Corman et al. 2020). However, other studies have found that the detection based on ORF1B-NSP14 had better detection effect than the detection based on RdRp (Chan et al. 2020). Chu et al*.* designed two detection methods targeting the ORF1b and N gene regions. It was suggested to employ the targeted N and ORF1b gene as screening and confirmation methods, respectively, because the targeted N gene had a considerably better sensitivity for the detection of clinical samples than the ORF1b gene (Chu et al. 2020). Some researchers had developed multiplex PCR methods that amplified two gene regions in the same PCR reaction, and the results were almost identical to the authorized detection systems by the Centers for Disease Control and Prevention, achieving rapid, accurate, and reliable detection (Tombuloglu et al. 2021). Interestingly, 31,421 SARS-CoV-2 samples showed that the gene region of the N-gene primers and probe targets was prone to mutation, which interfered with RT-PCR (Wang et al. 2020a, b, c).

Mutations in the target gene region can interfere with RT-PCR results, and false-negative results can occur when the viral load is below the detection limit. The positive detection rate is typically only 30% when testing on a large scale. And the viral load is mainly related to the collection time and sample type. Nasopharyngeal and oropharyngeal swab specimens were collected after the onset of symptoms, patients continued to shed SARS-CoV-2 virus with time, and the positive detection rate continued to decline (Kim et al. 2022). The positive rates of bronchoalveolar lavage fluid, nasal swabs, throat swabs, sputum, stool, and blood samples had been reported to vary widely (Connor et al. 2022; Wang et al. 2020a, b, c).

In summary, RT-PCR remains the “gold standard” for diagnosis due to its high sensitivity and specificity (Artika et al. 2022; Karlafti et al. 2023). However, it has limitations, such as susceptibility to RNA mutation, and high requirements for sample purity, reagents, machines, and professional expertise. False positives and false negatives are also potential issues (Khan et al. 2022). Nevertheless, RT-PCR based detection is still worthy of further improvement and exploration (Chavda et al. 2023).

### Loop-mediated isothermal amplification (LAMP)

Loop-mediated isothermal amplification (LAMP) is a novel nucleic acid amplification technology that allows for simple and efficient detection without the need for expensive thermal cycle machines (Cascella et al. 2022). As illustrated in Fig. 3B, LAMP employs four special primers that hybridize with six different regions of the target gene under constant temperature (65℃) and with the help of strand displacement DNA polymerase, to achieve isothermal amplification (Mori and Notomi 2009). Different primers designed for target genes leaded to different detection results, as shown in Table 2. A significant amount of magnesium pyrophosphate white precipitate was produced as a result of the DNA synthesis during the LAMP process, which could precipitate pyrophosphate ions and mix with magnesium ions in the reaction system. Therefore, turbidity analysis, fluorescence parameters, and colorimetry could be used to determine the detection results (Choi et al. 2022; Nawattanapaiboon et al. 2021; Notomi et al. 2000). To find SARS-CoV-2, many laboratories have developed the RT-LAMP approach. This approach involves reverse RNA into cDNA followed by LAMP isothermal nucleic acid amplification. The minimum virus detection limit was 20 copies, and the results can be obtained within an hour (Hu and Wang 2020; Yan et al. 2020). Jiang et al. (2020) compared RT-LAMP with RT-PCR in a clinical sample of 213 negative and 47 positive patients. The former showed high sensitivity and specificity compared to the latter, and the detection was twice as fast as the latter (Jiang et al. 2020). Combined with the indicator, RT-LAMP could intuitively observe the result through the color change of the solution (Yu et al. 2020). A research team had developed a detection method combined with RT-LAMP and nano-particle biosensor, which had achieved 100% sensitivity and specificity in testing collected clinical samples, which was an encouraging COVID-19 diagnostic candidate (Zhu et al. 2020). Additionally, a 40 min detection time of 4 copies/µL of SARS-CoV-2 RNA was achieved using an AuNP-based colorimetric technique in conjunction with Cas12a and RT-LAMP CLAP assay (Zhang et al. 2021b, a). Sherrill-Mix et al. 2021 developed LAMP-BEAC detection by combining RT-LAMP with molecular beacons. The customized polymerase and simple purification procedure reduced the cost of detection and could screen thousands of samples within a week (Sherrill-Mix et al. 2021). There are also many studies on LAMP as a diagnostic tool, such as EI-Tholoth et al*.*’ s new two-stage LAMP design for COVID-19 called the COVID-19-Penn-RAMP strategy, including recombinase isothermal amplification (RT-RPA) as its first stage and LAMP as its second stage. Lu et al. 2020a s testing of a LAMP based assay targeting the RdRp gene, etc.

**Table 2:** SARS-CoV-2 RT-LAMP tests in different laboratories

Target genes	LOD (copy/reaction)	Sensitivity (%)	Specificity (%)	Detection duration (min)	References
N, S and RdRp	118.6	94	90	20	Lu et al. (2020a, b)
N	100	100	98.7	30	Baek et al. (2020)
ORF1a	200 copies/μL	100	77	25	Torezin Mendonça et al. (2022)
ORF1b and N	500 copies/mL	91.4	99.5	30	Jiang et al. (2020)
ORF1a/b	20	100	100	60	Yan et al. (2020)
Nsp3	100	100	–	40	Park et al. (2020)

In summary, the LAMP technique is a highly reliable and low-cost diagnostic tool that can be used directly from patient specimens (Domnich et al. 2021). Overall, the detection time is a little less than RT-PCR. It has shown great potential for a variety of diagnostic settings and is particularly beneficial for developing and low-income countries, where it can significantly reduce testing time and improve the efficiency of virus screening. LAMP has also been integrated with mobile apps to provide highly accessible diagnostic tools for COVID-19 (Yang et al. 2020). However, designing and screening primers for LAMP can be challenging, and there is a risk of forming aerosol pollution during the experiment. Additionally, achieving optimal detection temperature and time presents significant challenges, and there are limitations in providing information for patients who have not fully recovered (D'Cruz et al. 2020; Zeng et al. 2020). Despite the quick development among those RT-LAMP based assays, cross-reactivity has prevented the commercialization of many methods.

### Clustered regularly interspaced short palindromic repeats (CRISPR)

**Fig. 4:**
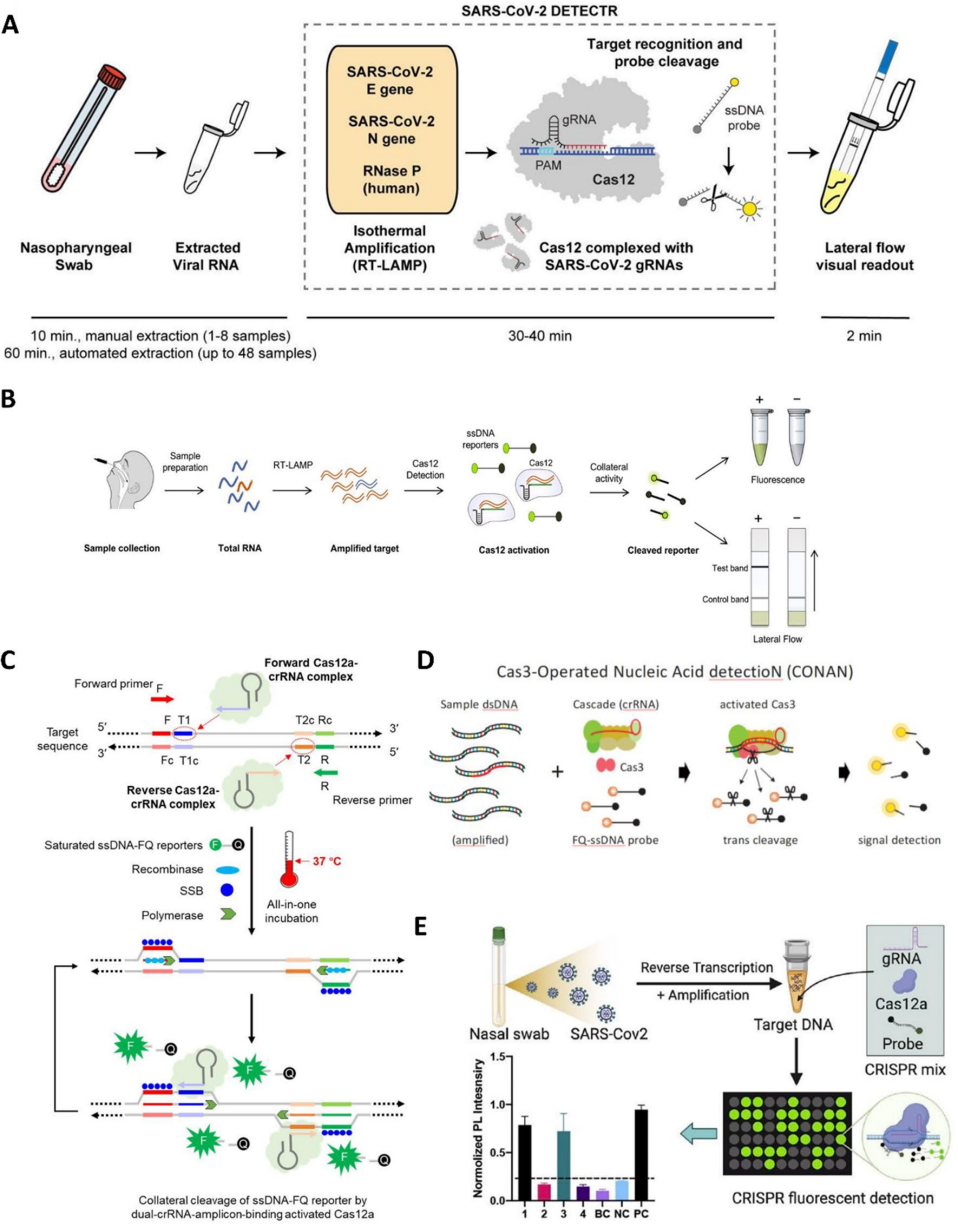
Flowchart of a CRISPR-based SARS-CoV-2 detection technique. **A** Workflow diagram for SARS-CoV-2 detection (Broughton et al. 2020). **B** Workflow of iSCAN detection assay (Ali et al. 2020). **C** Schematic of the AIOD-CRISPR assay system. **D** In vitro nucleic acid detection platform for CONAN shown schematically (Yoshimi et al. 2022). **E** CRISPR-FDS assay schematic for identifying SARS-CoV-2 RNA in clinical samples (Huang et al. 2020a, b)

The CRISPR system is a sequence of repeated nucleic acids found in bacteria as an immune defense mechanism against viral invasion (Torres-Ruiz and Rodriguez-Perales 2017). The system consists of guide RNA (gDNA) and Cas proteins, which binds to the target RNA/DNA sequence and instructs the Cas protein to identify and break down foreign nucleic acids (Karimian et al. 2019; Kellner et al. 2019). The system can also be used as “molecular scissors” to manipulate biological DNA/RNA, and has potential applications in clinical diagnostics (Chertow 2018; Li et al. 2019). Cas12a and Cas13a are the main CRISPR RNA enzymes used for target recognition and decomposition. When the target RNA or DNA sequence is bound by the guide RNA, Cas12a or Cas13a specifically decomposes the single-stranded target nucleic acid sequence, and then the non-specific endonuclease activity of Cas12a or Cas13a is activated, which splits the nearby non-target nucleic acid sequence and generates signals, providing ideas for the detection of specific nucleic acids (Kellner et al. 2019). By combining CRISPR-Cas system with other molecular assays, researchers have developed various SARS-CoV-2 detection techniques (Fig. 4). For the detection of SARS-CoV-2, Zhang and colleagues created a CRISPR-based SHERLOCK detection approach (Joung et al. 2020). The two primary components of the system were CRISPR-Cas13a nucleic acid identification and isothermal target nucleic acid amplification. Novel coronavirus detection had been successfully carried out in the range of 10–100 samples per microliter input with single-molecule sensitivity. Cas13a is not activated when the target RNA mismatch is greater than one, so with great specificity, it can quickly differentiate SARS-CoV-2 from other viruses. Although the dipstick test simplifies the operation and reduces the testing cost, the technique has not yet been tested in patients. Mammoth Biosciences’ DETECTR, which combined CRISPR-Cas12a-based technology with RT-LAMP, had the capacity to depict detection outcomes from clinical studies involving close to 100 individuals with COVID-19 infection and other viral respiratory illnesses. The specificity and positive predictive value of SARS-CoV-2 were 100% and 95%, respectively (Broughton et al. 2020). Similarly, Ali et al. 2020 iSCAN test was efficient and accurate for early diagnosis of COVID-19 carriers (Ali et al. 2020). Additionally, Ding et al. 2020 created an all-in-one dual CRISPR-Cas12a (AIOD-CRISPR) assay with a detection limit of 4.6–11 copies/µL that specifically targeted the cDNA of the N gene of SARS-CoV-2 (Ding et al. 2020). Additionally, some researchers have used fluorescence probes and CRISPR-Cas12a/gRNA complexes to detect target amplification sequences after standard PCR operations. This method has a limit of detection of 2 copies and is more accurate and sensitive CDC-approved quantitative RT-PCR detection results (Huang et al. 2020a, b). Recently, Yoshimi et al. 2022 developed a nucleic acid test based on Cas3a in vitro, known as CONAN, which showed high accuracy and specific recognition of single base pairs. The method detected 9 out of 10 positive samples and 1 out of 21 negative samples, indicating its potential as a sensitive and accurate diagnostic tool (Yoshimi et al. 2022).

In summary, CRISPR-based SARS-CoV-2 offer a promising alternative to traditional diagnostic methods, with potential advantages in terms of cost, speed, and ease of use. However, further validation is necessary to ensure their accuracy, specificity, and reliability, and to address potential issues such as false positives or false negatives. Despite these challenges, the use of CRISPR technology in diagnostic applications represents an exciting new frontier in the fight against infectious diseases. The effectiveness of these methods needs to be carefully tested in the clinic. Moreover, the comparison of major molecular biological diagnostic methods for the detection of SARS-CoV-2 are shown in Table 3.

**Table 3:** Comparison of major diagnostic methods for the detection of SARS-CoV-2

Technology	Manufacturer	Delivery time of results	Comments	References
RT-PCR	Roche	3–4 h	One of the first available tests	Corman et al. (2020)
RT-PCR	Bosch	2.5 h	Fully automated, POC, testing samples for COVID-19 and 9 other pathogens of the respiratory tract	*Bosch Global, (2020)
RT-PCR	Mesa Biotech	30 min	Qualitative with a visual read of results, POC, using throat and nasal swabs, targeting N protein	**Mesa Biotechnology, (2020)
RT-LAMP	Abbott Laboratories	5–13 min	Fast delivery of the positive results in 5 min, small size that can be used in clinics	#Abbott Laboratories ^(^2020a)
RT-LAMP	Sanya Peoples Hospital, Sanya, Hainan	1 h	Higher sensitivity compared with established RT-LAMP, amplification in one step and single tube	Zhu et al. (2020)
CRISPR	–	1.2 h	Simple, efficient and improved specificity, but risk of contamination	Broughton et al. (2020)
CRISPR	–-	20–50 min	Low level instrumentation is required, higher possibility for medium mutation detection	Liang et al. (2021)

*Bosch Global, 2020. URL https://www.bosch.com/stories/vivalytic-rapid-test-for-covid-19/

**Mesa Biotechnology, 2020. URL https://www.mesabiotech.com/coronavirus

^#^Abbott Laboratories, 2020a. URL https://www.abbott.com/corpnewsroom/product-and-innovation/detect-covid-19-in-as-little-as-5-minutes.html

### Whole-genome sequencing of SARS-CoV-2

The SARS-CoV-2 is primarily transmitted through respiratory droplets and close contact. Whole genome sequencing (WGS) data are used to study pathogen transmission and microevolution, enabling the determination of direct transmission between donor and recipient. Viral RNA was amplified by PCR, sequencing library was constructed, a high-throughput sequencing template was prepared, and WGS was performed (Nasir et al. 2020).

When SARS-CoV-2 is spread to another area, local outbreaks occur, requiring urgent testing and tracing for initial containment of infected persons. Genome sequencing can help analyze pathogen transmission (Gilchrist et al. 2015). WGS of SARS-CoV-2 provided additional data to complement routine diagnostic testing. Viral WGS helps to analyze the spread of pathogens by determining the transmission relationship between the virus and the patient to reconstruct the transmission network. At the same time, it can also monitor the emergence of dangerous variants and help to study the adaptation of pathogens in the host, which also has important implications for vaccine development (Jain M et al. 2020). The release of the complete genome sequence in January 2020 facilitated the development of RT-PCR assays for the detection of SARS-CoV-2, which has become a diagnostic criterion during the ongoing COVID-19 pandemic (Seth-Smith et al. 2019; Van Kasteren et al. 2020). Long read sequencing using Oxford Nanopore Technology (ONT) captures reverse transcribed SARS-CoV-2 genome sequence by PCR amplification and then performed high-throughput sequencing. ONT relies on a nanoscale protein pore, each used for DNA or RNA sequencing. ONT has many advantages, such as portable equipment, low price, and flexible and scalable rapid sequencing analysis (Bull et al. 2020). To address the issue of ONT sequencing accuracy and to evaluate its effectiveness in SARS-CoV-2 genomic analysis, amplicon-based nanopore and short-read WGS were performed on matched SARS-CoV-2 positive patient samples and synthetic RNA controls (Jain et al. 2016). Further evaluation of the suitability of ONT is expected to be applied to many more scenarios.

### Others

#### Cartridge based nucleic acid amplification test (CBNAAT)

**Fig. 5:**
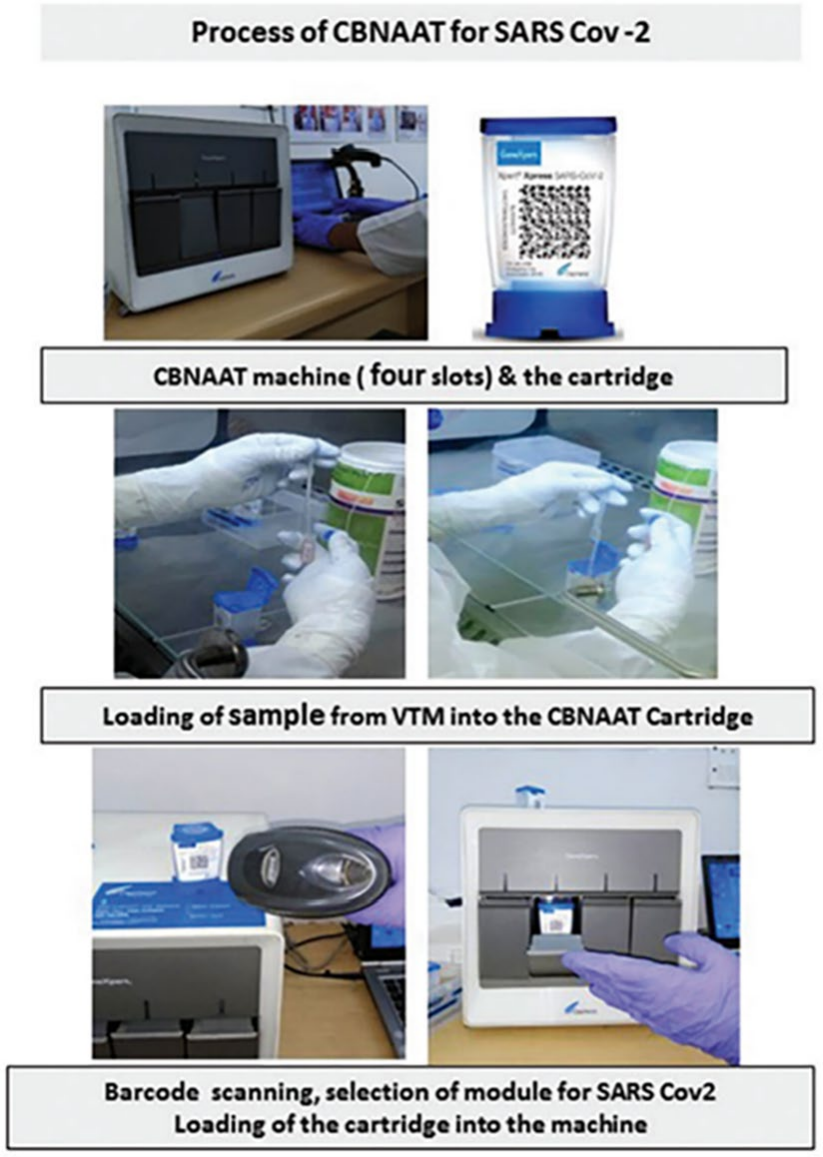
Workflow in CBNAAT platform for detection of SARS-CoV-2 (Gogoi et al. 2021)

CBNAAT, an automated molecular diagnostic technology approved by the WHO, is used for the diagnosis of tuberculosis in children (Agarwal et al. 2022). Due to the rising number of SAR-COV-2 infections, the Indian Council of Medical Research has developed and approved the use of CBNAAT assay for SAR-CoV-2 detection (Gogoi et al. 2021). The CBNAAT assay utilizes real-time quantitative PCR to integrate SAR-CoV-2 sample preparation, RNA extraction, amplification, and target sequence detection in about 45–60 min. Nasal lotion specimens or nasopharyngeal swab samples were obtained in viral transport media (VTM), transported to the laboratory under cold chain, mixed thoroughly in an inverted tube for several times, and then transferred to the sample chamber of an Xpert Xpress SAR-CoV-2 cartridge. Finally, real-time quantitative PCR was performed in the GeneXpert instrument system (Fig. 5). The likelihood of cross contamination is very low, and the operation is simple and time-efficient. However, it requires a continuous power supply from the machine, and false negative results may occur if the selected S and N2 target genes have mutations.

#### Gene chip

Gene chip technology utilizes the principle of nucleic acid hybridization to detect and quantify sequence molecules. For COVID-19, CapitalBio Corporation, in collaboration with Tsinghua University and Sichuan University, has designed a kit using a constant temperature amplification chip method that can detect several common respiratory viruses, including SAR-CoV-2 and influenza virus, with high accuracy. However, the gene chip testing method is prone to false positive results and carries the risks of aerosol contamination and infection when adding labeled samples to the gene chip. Nonetheless, gene chip technology is an efficient testing method for detecting respiratory viruses.

#### ID NOW

The ID NOW test kit is one of the most popular point-of-care molecular detection technologies in the United States, due to its portability and high efficiency, which based on the nicking enzyme amplification reaction (NEAR) technology, allowing to give a qualitative result (positive, negative, uninterpretable) for the detection of the RdRp gene segment of SARS-CoV-2. It is controlled by constant temperature to achieve large RNA amplifications that can be detected within minutes. Samples obtained from 72 patients infected with SAR-CoV-2 were observed to have sensitivity of more than 90% and specificity of 100% for ID NOW (Srivastava et al. 2022). In 259 cases of different age groups, the sensitivity, specificity, and positive and negative predictive values of ID NOW were all above 90% (Pattnaik et al. 2022). However, even though ID NOW has strong positive and negative predictive values, it is currently only authorized by the FDA for emergency use. Additionally, the test requires less than an hour for sample transportation, which may not be applicable in some regions.

### Droplet-based digital PCR (d-PCR)

Droplet-based digital PCR (d-PCR) method, which has been used for new variants of the COVID-19 virus, is one of the most accurate systems for effectively detecting viral infections with hypersensitivity and specificity.

Throughout the COVID-19 pandemic, concerns have arisen regarding the recurrence of independent variants of the SARS-CoV-2 virus, known as variants of concern (VOCs). These variants are characterized by their increased transmissibility, virulence, or reduced susceptibility to antibodies acquired through prior infection or vaccination. The presence of SARS-CoV-2 RNA in body fluids, such as feces, has made it easily detectable in wastewater (Wang et al. 2020a, b, c). As a result, monitoring wastewater quality through sequencing has become a new method of exploring the spread of SARS-CoV-2 variants (Caduff et al. 2022). Sequencing of SARS-CoV-2 in wastewater has already been used to detect novel mutations in the San Francisco Bay Area (Crits-Christoph et al. 2021). The α, β, and γ variants have spread globally, with the α variant being the most prevalent in Europe. As the number of reported infections has increased, the α variant has become the primary target for surveillance. Tracking the introduction and spread of VOCs requires whole-genome sequencing of clinical samples, and PCR-based assays have become increasingly common due to the need for rapid, inexpensive, high-throughput, and easily interpretable methods. The d-PCR enables absolute quantification of nucleic acids in samples (Hindson et al. 2011). And increasingly used to quantitatively detect COVID-19 (Gerdes et al. 2016). It is used for molecular analysis of next-generation sequencing-related microRNAs in clinical and research applications (Ma et al. 2013). The principle of d-PCR is to limit the dilution of the reaction system containing nucleic acid molecules and amplify the target nucleic acid sequence into millions of uniform-sized nanoliters or picoliters of independent reaction zones. The presence or absence of the terminal signal is used to determine the outcome (Hindson et al. 2011). The advantages of d-PCR include high sensitivity, high accuracy, strong resistance to inhibitors, an increased signal-to-noise ratio in sample distribution, and dilution of background signals to detect targets with low abundance.

Moreover, d-PCR has been found to be more sensitive than RT-PCR in the diagnosis of COVID-19. Originally designed for use in microbiological diagnostics, specifically for viruses. After the viral load of an infected individual is determined by the d-PCR diagnostic assessment, the infectivity of the virus is further confirmed. Now d-PCR is being utilized for COVID-19 diagnosis (Falzone et al. 2021). The d-PCR scheme method works by diluting and distributing the reaction mixture into the nanodroplets created from an oil and water emulsion, and then the volume of the droplets is determined through microscope imaging. The accuracy range of d-PCR can be determined through threshold analysis of the image using image software (Pinheiro et al. 2012). Because of its practicality and low cost, d-PCR is helpful for the early treatment of SARS-CoV-2 and the prevention and control of virus transmission (Suo et al. 2020). To compare the sensitivity of RT-qPCR and d-PCR to detect low viral load in COVID-19 positive patients. Researchers analyzed two blind throat swabs taken from two patients who tested positive and negative for COVID-19. By comparing the two methods, they concluded that d-PCR is more sensitive than RT-qPCR, however, it is also more expensive and takes longer. Overall, d-PCR takes about 15% more time and cost than RT-qPCR. This method provides a complementary approach to clinical sequencing analysis and enables earlier detection and inference of the transmission rate of COVID-19 variants.

## Immunological assays

Immunodetection is to detect viral proteins (antigens) in the patient’s body through serum, saliva, etc., or specific antibodies to determine whether the patient is infected by a virus, which is also the biggest difference between immunodetection and nucleic acid testing.

Following infection with SARS-CoV-2, the body shows a significant inflammatory response, and supplementing nucleic acid testing with immunological assays can reduce the risk of false negatives and lower the exposure risk for healthcare workers during respiratory specimen collection. Immunodetection methods can quickly identify infected patients, particularly in cases where RT-PCR results are not immediately available, enabling early intervention and treatment and reducing the spread of the virus. These methods have been widely adopted in many countries (Lou et al. 2020; To et al. 2021).

**Fig. 6:**
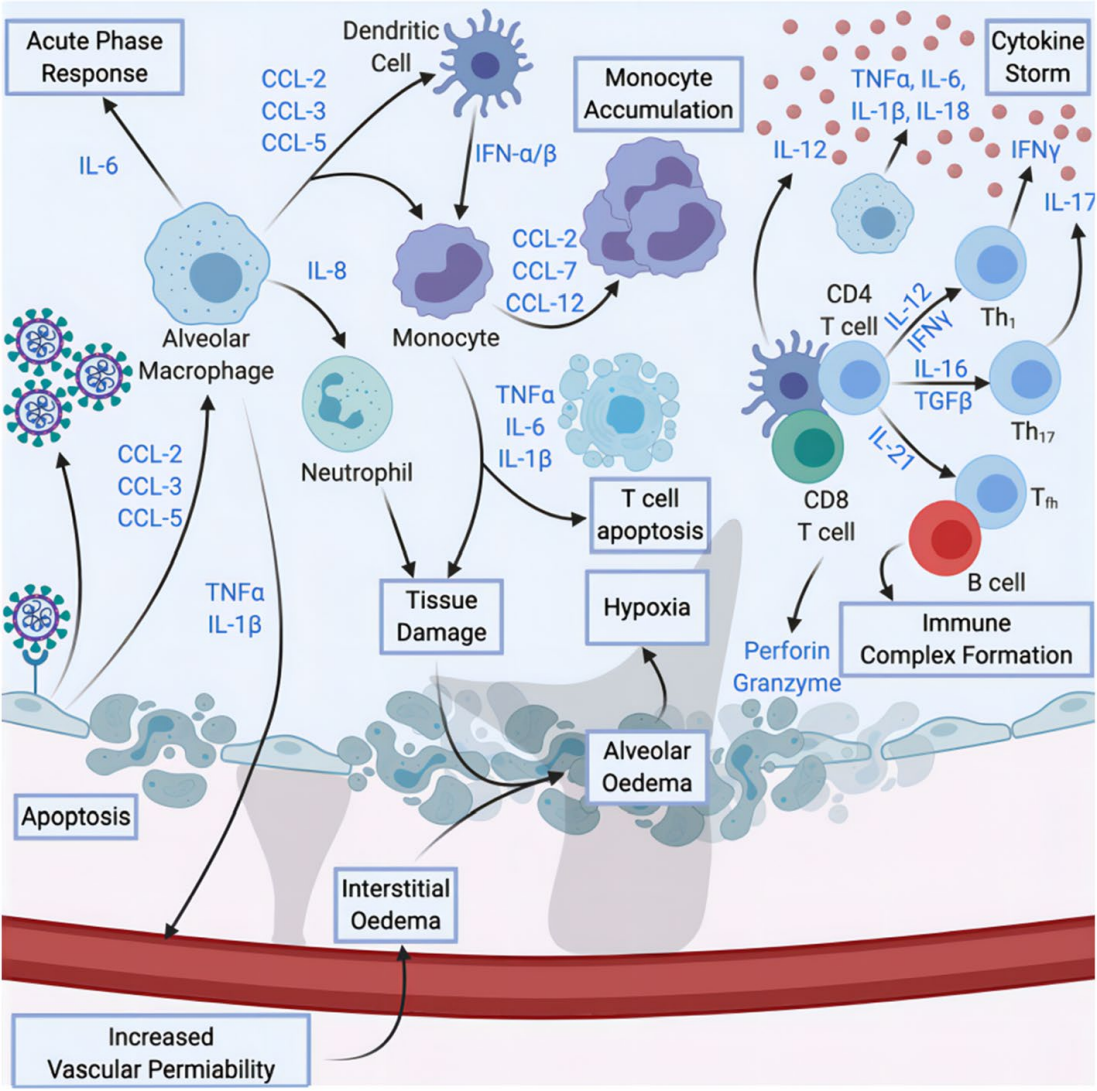
The cytokine storm caused by immunopathology of COVID-19 patients (Anka et al. 2021)

Infected cells produce cytokines that attract macrophages and other immune cells, thereby increasing vascular permeability and triggering acute phase responses. Infected cells die leading to tissue damage or necrosis, and increased interstitial fluid causes edema and hypoxia. Innate and adaptive immune that hyperactivated responses induces cytokine storm. These factors ultimately lead to acute respiratory distress syndrome (ARDS) (Anka et al. 2021) (Fig. 6). Immunological detection methods include colloidal gold immunochromatography, enzyme-linked immunosorbent assay, chemiluminescence immunoassay, magnetic particle chemiluminescence, up-conversion luminescence immunochromatography, quantum dot fluorescence immunochromatography, immunofluorescence detection, direct fluorescent antibody detection, etc. The similarities and differences of the main immunological-based methods are shown in Table 4.

**Table 4:** Comparison of analytical performances of immunoassay-based COVID-19 diagnostic methods

Methods	Target analyte	Sample type	Clinical sensitivity (%)	Specificity (%)	Test time (min)	Advantages	Disadvantages	References
GICA	IgM, IgG or IgM/G	Blood	79.7% ~ 86.43%	99.57% ~ 100%	15–20	Easy to standardize, fast, low equipment cost	For clinical auxiliary	Li et al. (2021b, a); Mekonnen et al. (2021)
ELISA	IgM, IgG, IgA, nucleocapsid or total antibodies	Blood	Around 86% (total antibodies: 94%)	99%	60–180	Fast, reduce the risk of infection for testing personnel, low equipment cost	Not suitable for the identification of the active cases	Mekonnen et al. (2021); Peterhoff et al. (2021); Beavis et al (2020)
CLIA	IgM, IgG or IgM/G	Blood	Around 92% (CLIA-RBD:96%)	99%	30–60	No enzymes catalysis, high specificity, wide linear range, fast	The influence of the environment on the detection is relatively large; easy to cause errors	Peterhoff et al. (2021)

### Colloidal gold immunochromatographic assay (GICA)

**Fig. 7:**
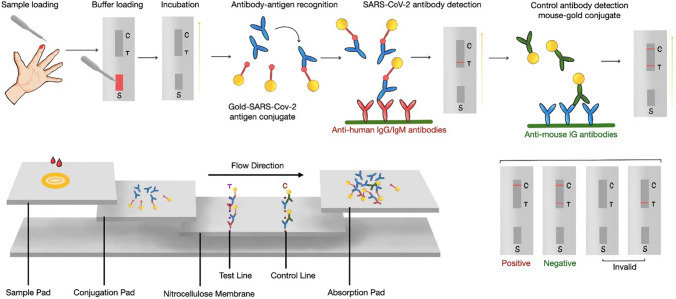
Based on the principle of specific binding of IgG (IgM) antibodies. A–E, schematic diagram of the detection process colloidal gold kit. **A** Blood from the patient’s fingertip and drop to the sample pad. **B** Dropwise saline buffer for dilution the sample. **C** Wait for incubation. **D** Antibody-antigen recognition. **E** SARS-CoV-2 antibody detection. **F** Complex color reaction on the control line. **G** Schematic diagram of the colloidal gold kit consisting of a sample pad, a binding pad or a binder release pad, a nitrocellulose filter membrane (NC filter) and a water absorbing pad. SARS-CoV-2 antigen is used in the control line and the mouse anti-human IgG (IgM) is placed in the test line. **H** The detection result is valid only when the control line is colored

GICA uses colloidal gold as a tracer and relies on the specific binding and color development of antigen–antibody reactions. Figure 7 illustrates the principle of specific binding of IgG (IgM) antibodies in GICA. This technique is easy to standardize, commercialize, and operate, making it widely used in clinical diagnosis for COVID-19. Compared with other serological test methods, GICA is sensitive, specific, time-saving, material-saving, and simple to operate. However, it is relatively insensitive and cannot quantitatively detect antibodies, and is mainly used for aiding rapid screening (Li et al. 2021b, a). Through subgroup analysis, Zhang found that chemiluminescence immunoassays and GICA were more sensitive to IgG and IgM than ELISA. Similar detection methods are used for SARS and SARS-CoV-2. GICA is considered the preferred method for detecting anti-SARS-CoV-2 IgG and IgM due to its high efficiency and simple operation (Zhang et al. 2021b, a). However, patients with autoimmune diseases may have false positive results using GICA tests, as elevated serum rheumatoid factor levels may lead to false positives (Xiao et al. 2021). Additionally, GICA is considered a highly sensitive and specific detection method for monitoring the total level of antibodies after vaccination, which can help to evaluate the vaccine’s protection rate and effectiveness (Hu et al. 2021; Liu et al. 2021; Li et al. 2021b, a; Pan et al. 2020; Ye et al. 2022).

### Enzyme-linked immunosorbent assay (ELISA)

**Fig. 8:**
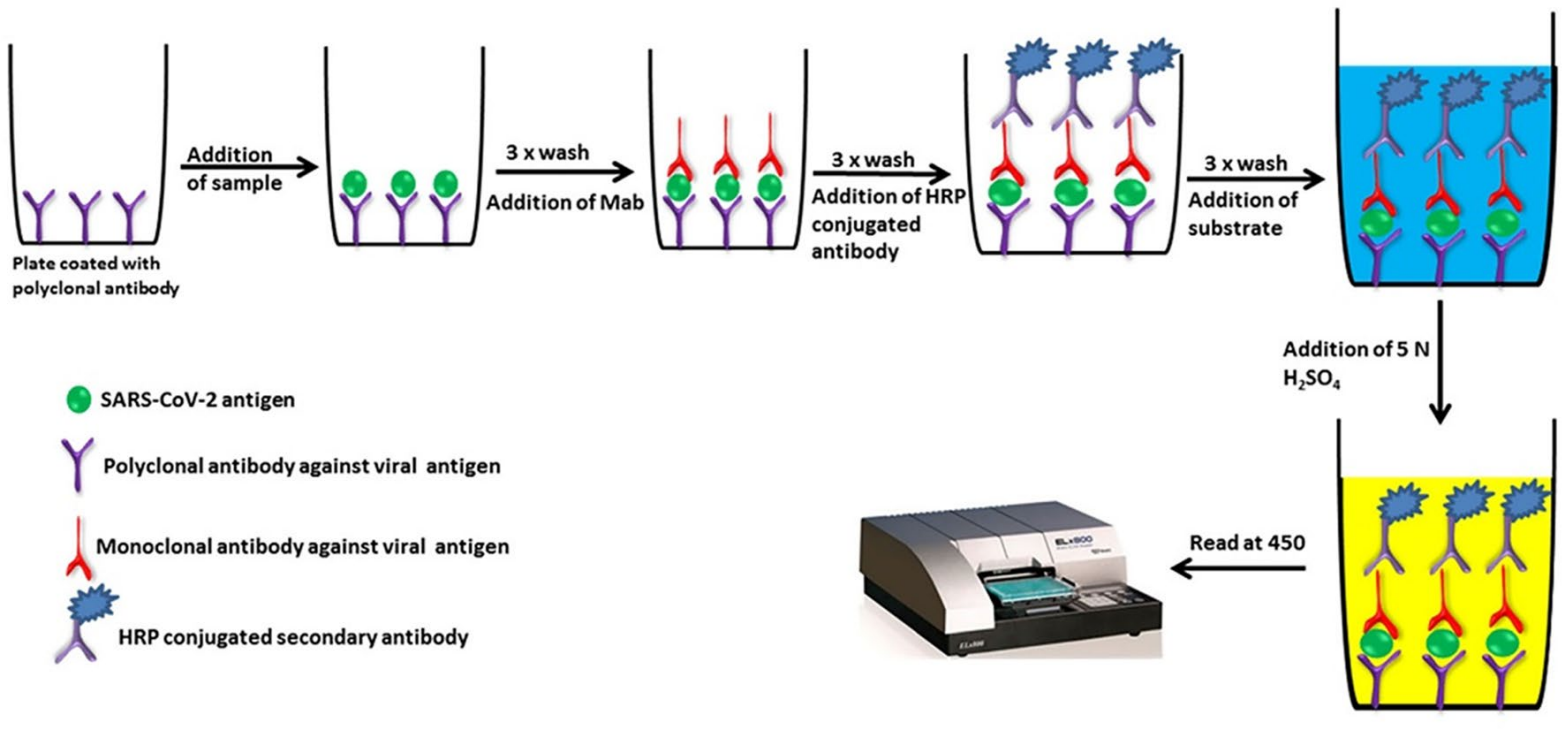
Schematic diagram of ELISA (Filchakova et al. 2022). Special solid-phase carriers are used to maintain the immune activity of antigens and antibodies, and enzymes are used to link antigens and antibodies to form enzyme markers. Enzyme-labeled antibodies or antigens have both the immunological and catalytic activity of the enzyme. The addition of the corresponding substrate is enzymatically catalyzed into a colored product. The color shade allows quantitative analysis of the measured sample. The efficient catalytic nature of enzymes greatly increases the sensitivity of this method

A useful tool for determining the presence or absence of a specific target protein. ELISA is a microplate-based assay technique designed to detect and quantify substances such as peptides, proteins, antibodies and hormones. The test can be qualitative or quantitative, and the process usually takes 1 to 1.5 h. Microplate pores are usually coated with a viral protein. Antiviral antibodies in patient samples will bind specifically, and the bound antibody-protein complex can be examined with additional tracer antibodies to produce colorimetric or fluorescence-based readings (Carter et al 2020) (Fig. 8). The University of Chicago conducted tests on samples from COVID-19 patients and non-COVID-19 patients, and after ELISA testing, statistical analysis and PCR validation showed that ELISA has good sensitivity for IgG testing (Beavis et al. 2020).

Okba tested an ELISA for detecting IgA and IgG antibodies using the Spike protein S1 antigen. Data analysis revealed that both ELISAs displayed some cross-reactivity with other human coronavirus-positive patients’ samples. In terms of detection, IgA ELISA showed higher sensitivity, while IgG ELISA demonstrated higher specificity.

Theano and Vana mixed the receptor-binding domain (RBD) and Spike protein S2, nucleocapsid, and after numerous experiments, they developed an “in-house” serological ELISA, which had a sensitivity and specificity of 75% and 95% for detecting IgM, respectively. Peterhoff conducted comparative experiments on the serum of patients infected with seasonal coronaviruses, SARS-CoV-2, and control groups. They recorded IgM, IgA, and IgG antibody responses to these antigens. After a series of evaluations, RBD ELISA has great potential in assessing infection and vaccination (Lagousi et al. 2021; Peterhoff et al. 2021). In addition, ELISA antibody titers are thought to have a correlation with neutralizing antibody levels. S/Co (absorbance unit) values can accurately estimate ELISA antibody titers, and combined with methods that utilize chemiluminescence, can provide an accurate, rapid, and cost-effective method for identifying anti-SARS-CoV-2 antibody titers in sample serum for the treatment of COVID-19 infection (Salazar et al. 2020; Krähling et al. 2021). MacMullan has developed a convenient, inexpensive and efficient, non-invasive antibody, the kit using only a saliva sample. This method eliminates the need for professional physicians to draw blood to collect serum samples, thereby reducing the risk of exposure of medical personnel to potentially infectious environments, and is suitable for widespread, frequent testing to assess seroprevalence in susceptible species or to screen hosts or intermediate hosts (MacMullan et al. 2020; Wernike et al. 2021).

### Chemiluminescence immunoassay (CLIA)

CLIA is a detection method that combines a highly sensitive chemiluminescent reaction with a highly specific immune response. Chemiluminescence technology involves the formation of an excited state following a specific substance undergoing a chemical reaction. When this excited state returns to the ground state, it releases photons, and the luminous intensity of the reaction can be detected by the instrument based on the quantum yield of the luminescent reaction.

In CLIA, immunolabeling technology is used to label the substrate or catalyst (enzyme or inorganic catalyst) of the chemiluminescent reaction on a pre-prepared specific antigen or antibody. This approach enables the detection of target molecules with high sensitivity and specificity. The concentration of the analyte relates to the chemiluminescence intensity through the immune response bridge to achieve quantitative detection of the analyte (Cinquanta et al. 2017; Li et al. 2020). Sekirov used six kinds of CLIA to collect and analyze samples from symptomatic infected patients 14 days after symptom onset, and compared with PCR results, it found that CLIA had high specificity and sensitivity, but the sensitivity was significantly reduced after 14 days (Sekirov, et al. 2021). Liu conducted a controlled study of 270 healthy serum samples and 206 patient serums, and used chemiluminescence particle immunoassay (CMIA) to detect IgM and total Ab against SARS-CoV-2, which implied that CMIA was significantly more sensitive to total Ab detection than IgM. Combined with CLIA testing in clinical applications can effectively reduce the rate of misdiagnosis (Liu et al. 2020a, b; Shao et al. 2020; Grenache et al. 2021).

## Pulmonary imaging detection

COVID-19 primarily spreads through respiratory droplets, and lung lesions reflect its pathological changes. In the early stages, multiple small patches and interstitial changes are observed. The outer lung band is obvious, and then develops into a multi-lobular ground glass shadow and infiltrating shadow. The lung imaging can detect COVID-19 at an early stage with high sensitivity, and quickly detect lung lesions. Compared with other methods, lung imaging equipment is convenient to operate, and low examination costs, making it widely used in many countries.

**Fig. 9:**
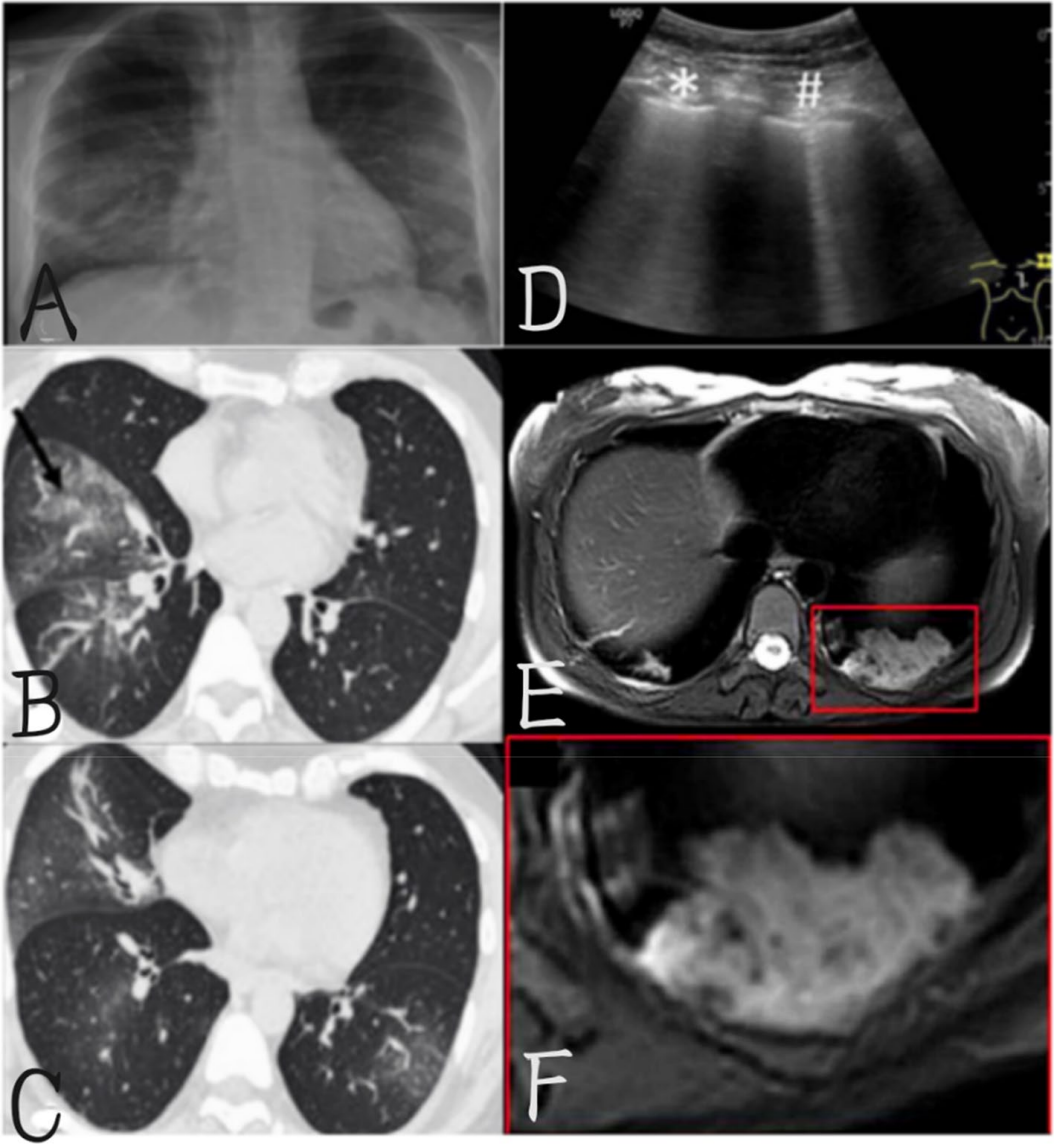
**A** The frontal X-ray showed bilateral blurry, mainly involving the middle and lower lungs. **B**, **C** Chest CT showed no contrast showing multiple ground-glass and “crazy pavement” images in the right middle and lower lobe (Pontone et al. 2021). **D** A lung sonogram of a patient with COVID-19. Corresponding diffuse alveolar damage was also detected in the histopathological findings of a patient with SARS-CoV-2 infection, which was described as multifocal frosted glass opacity with irregular paving patterns on CT diagnosis and is described as a typical finding in the early stages of the disease. The sonography associated with these CT findings is formed by multiple B-lines that are unevenly distributed bilaterally and are clearly focused on the normal area (Nouvenne et al. 2020). (**E,** **F**) shows an MRI of COVID-19 with a corresponding focus image of consolidation in the left lower lobe (X3) (Nissan et al. 2020)

Lung imaging tests include chest X-ray, computed tomography (CT), lung ultrasound (LUS), and magnetic resonance imaging (MRI). A comparison of the four lung imaging methods is shown in Table 5, and the representative images are shown in Fig. 9.

**Table 5:** Comparison of four pulmonary imaging detection

Method	Advantage	Disadvantage
X-ray	Simple equipment and low cost and ease of decontamination	Early slow sensitivity and the equipment is easy to be polluted
CT	High efficiency and early hypersensitivity	Poor specificity and high price
LUS	Low cost, radiation free and hypersensitivity	Specific disinfection difficulty
MRI	Auxiliary diagnosis	Slow imaging and low efficiency

### X-ray

**Fig. 10:**
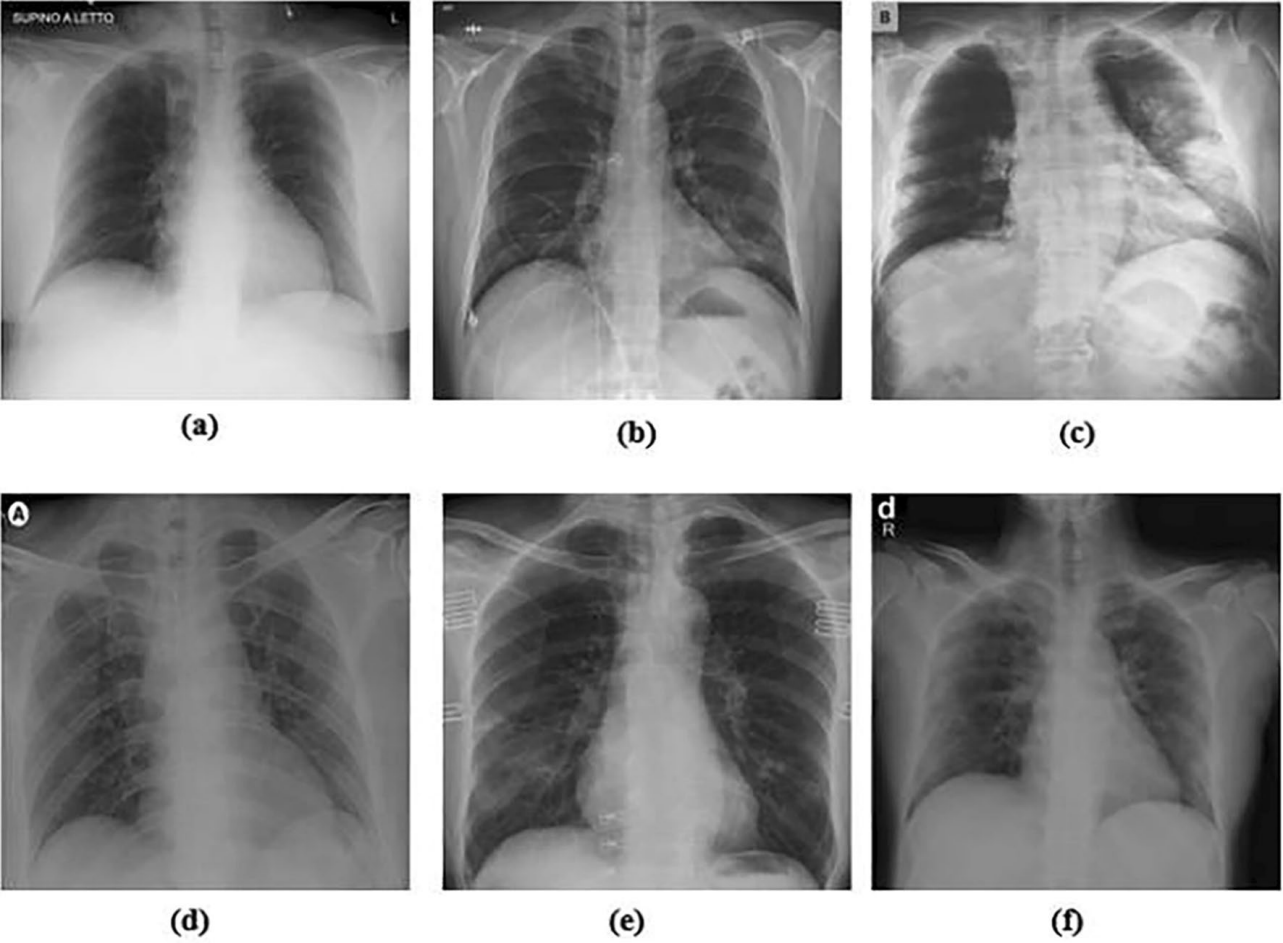
**A** Cardio-vasal shadow within the limits. **B** Increasing left basilar opacity is visible, arousing concern about pneumonia. **C** Progressive infiltrate and consolidation. **D** Small consolidation in right upper lobe and ground-glass opacities in both lower lobes. **E** Infection demonstrates right infrahilar airspace opacities. **F** Progression of prominent bilateral perihilar infiltration and ill-defined patchy opacities at bilateral lungs (Ozturk et al. 2020)

Chest X-ray (CXR) images have been used to initially diagnose COVID-19 by detecting abnormalities in lung (Fig. 10). CXR examination can reveal characteristic findings in the lungs related to COVID-19 (Ozturk et al. 2020). The mechanism of action is to collect all possible images of previous COVID-19 by using the generation adversarial network (GAN) to generate more images from previous COVID-19 cases and furthermore detect the disease with the higher accuracy from the available CXR images. CXR may not be sensitive enough to detect these changes in the early stages of infection. (Abd Wahab et al. 2022). For example, CT scan can more easily recognize hazy opacities as hairy glass opacity (GGO), which may appear as occult or subtle hazy opacity on the CXR. Opacification in the lower right airspace was observed in a COVID-19 patient, one-third of the study patients could detect nodular opacities in the left lower lung region (Kong and Agarwal. 2020). It also been found that GGO and parenchymal lung lesions, interlobular septal thickening, and air bronchography were common in the lungs of patients with COVID-19 (Kanne et al. 2020). The CXR in the frontal lobe was blurry bilaterally, and mainly involving in the lower or middle regions of the lung. Another observation is the involvement of both lungs with peripheral focal or multifocal GGO in 50 of 75% of patients. The primary disadvantage of CXR analyses is their inability to capture early lung changes in COVID-19, because they are not sensitive enough to detect GGO at an early stage. The CXR is not as sensitive as the CT scan. Due to its availability and ease of decontamination, it is readily available as a first-line treatment. Deep learning models, such as convolutional neural network (CNN), a learning network which have the ability to represent learning and to simulates the construction of biological visual perception mechanism, have been used to detect COVID-19 in lung imaging and are highly sensitive. Therefore, the diagnosis is very accurate, so a well-trained deep learning model can focus on points that the human eye does not pay attention to, and may be used to reverse this perception of early low sensitivity in CXR.

### Computed tomography (CT)

The COVID-19 virus can cause infection in the upper respiratory tract and lungs. Therefore, chest CT scans can be used for routine diagnosis of COVID-19, especially high-resolution computed tomography (HRCT), which can reconstruct various layers of tissue structures at any depth or angle. Enabling the identification of minimal changes. Unenhanced HRCT obtained during a single breath hold is then subjected to reconstruction and transmission of CT images for subsequent interpretation and diagnosis. Different stages during COVID-19 have their own characteristics in CT performance. In the early stages (0–4 days), GGO abnormalities are present. The progressive stage (5–8 days) is characterized by an increasing number and scale of GGO. The peak stage is characterized by more extensive lung involvement, and dense consolidation that is slowly reabsorbed. The final absorption stage shows signs of repair in the lungs (Abd Wahab et al. 2022). CT scan of the non-contrast agent chest shows multiple GGO and a “crazy paving” pattern in the lower and middle lobes on the right. A middle-aged man with febrile diarrhea was admitted, and a nasopharyngeal swab confirmed that the patient was positive for SARS-CoV-2. The chest radiograph showed mild bilateral shadows, mainly involving the middle and lower lung areas, suggesting interstitial inflammatory involvement. No pleural effusion was found. The chest CT contrast scan on the same day showed multiple GGO. Four days later, the patient’s condition worsened, and the breathing became more difficult. Follow-up CT scans showed an increase in the degree of GGO mixed into the new consolidation area of the lower lobe (Pontone et al. 2021). A chest CT scan after contrast injection revealed the presence of a lung abscess in the left upper lobe. Comparing the previous CT scans showed that the GGO and consolidation regions were obviously reduced. A comparison of the sensitivity of chest CT with RT-PCR showed that the accuracy of chest CT scan for COVID-19 was 98%, while that of RT-PCR was only 71% when 51 patients were tested within 3 days (Fang et al. 2020). Studies have also found that a positive diagnosis of COVID-19 was confirmed by chest CT scan 3 days earlier than RT-PCR, Chest CT scans are now extensively used as an adjunct tool for COVID-19 diagnosis in China. However, chest CT scans also have poor specificity and expensive shortcomings, so their use is not very common.

### Lung ultrasound

The evolution of LUS results can be used to monitor the progression or regression of lung injury quantitatively and effectively (Hussain et al. 2020). LUS scans key areas using two types of portable ultrasound instruments, and serial images are used for pattern assessment, marking, saving, and archive clips (Abd Wahab et al. 2022). LUS findings in a COVID-19 patient, with corresponding diffuse alveolar damage detected in histopathological findings. In a patient with SARS-CoV-2 infection, which is described in CT diagnosis as multifocal frosted glass opacification with irregular paving patterns and described as a typical finding in the early stages of the disease. The ultrasonography associated with these CT findings is formed by multiple B-lines that are unevenly distributed bilaterally, which are clearly focused on the normal area LUS is a non-invasive, portable, and patient-friendly method that allows only a small contact area at the bedside and may yield better results in special situations or intensive care units (ICU) (Dudea 2020). It is highly specific in detecting lung involvement and monitoring lung changes in COVID-19 patients. Moreover, LUS can be performed even in the patient’s home, reducing waiting times for emergency room CT scans. However, the downsides of the LUS include the need for close contact between the device and patients during testing, increased the risk of infection for the inspector due to prolonged exposure to the environment, and difficulty in thoroughly cleaning and disinfecting the transducers. Despite the high sensitivity of LUS for COVID-19 pneumonia, the lungs do not exhibit characteristic signs associated with SARS-CoV-2 infection. All abnormal signs of COVID-19 pneumonia are identical to those seen in other interstitial and alveolar interstitial lung diseases (Campagnano et al. 2021).

### Magnetic resonance imaging (MRI)

Compared with other respiratory diseases, COVID-19 carries a higher risk of neurological disease, and magnetic resonance imaging (MRI) can provide valuable diagnostic information for COVID-19 patients who have central nervous system symptoms. MRI can be used to assist in the diagnosis of COVID-19 complications (Filatov et al. 2020). Additionally, lung gas MRI has several advantages over the commonly used lung CT imaging methods, including non-ionizing radiation and the ability to quantitatively assess pulmonary ventilation function, microstructure, and Qi-blood exchange function. Which makes it has great advantages in long-term follow-up of patients who have been discharged from hospital after COVID-19. In addition, through long-term follow-up monitoring, MRI can also evaluate whether the lung function damage caused by COVID-19 is a permanent change. It plays a crucial part in the late review stage of COVID-19 discharged patients (Giacomelli et al. 2020). An MRI of COVID-19 patients, corresponding focused image of the lower left lobe consolidation (Nissan et al. 2020).

## Biosensors

**Fig. 11:**
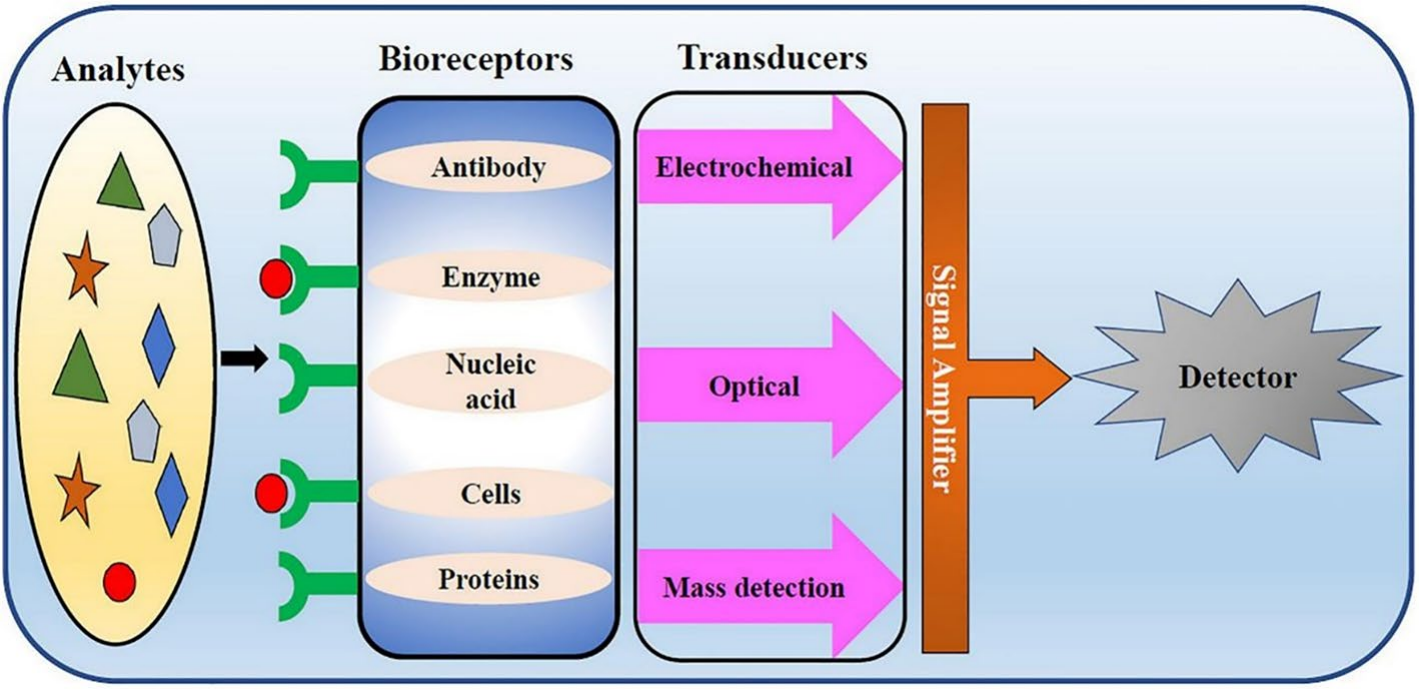
General structure of biosensor (Misra et al. 2022)

Biosensors are interpretive devices that can convert biological signals into electrical signals. They play a crucial role in early disease prevention due to their advantages, such as portability, speed and no need for intensive training (Mobed and Sepehri 2021). It provides a specific and rapid auxiliary method for traditional diagnostic analysis (Saylan et al 2017). Biosensors include three main elements: biological receptors, sensors, and signal processing systems (Perumal and Hashim 2014). Bioreceptors are biological elements that can recognize biometric molecules such as microorganisms, antibodies or nucleic acids and generate response signals. The second component, a sensor, converts the interaction between a bioreceptor and a biometric molecule into a measurable electrical signal. The third important part is the signal processing system, which receives the electrical signals from the second part of the sensor and can amplify them through an electronic display system to read and process the signals (Abid et al. 2021) (Fig. 11). Biosensors can be used in clinical diagnostic tests, treatment process monitoring, fermentation industry, and electrical treatment of water and food (Saylan et al. 2019). There are four types of biosensors based on the technology produced, namely optical biosensors, electrochemical biosensors, piezoelectric biosensors, and thermal biosensors (Samson et al. 2020). Optical biosensors are designed to measure changes in optical properties, including reduced or increased light, when biological receptors and biometric molecules interact (Garzón et al. 2019; Masson 2017). Electrochemical biosensors can monitor changes in the surface charge of sensors (Walker et al. 2021). Piezoelectric biosensors are promising viral biosensors based on the measurement of resonant frequency changes caused by changes in biometric molecular mass (Saylan et al. 2019). Thermal biosensors convert signals by measuring heat changes occurring during biochemical recognition using thermistors (Ramanathan and Danielsson 2001; Ramanathan et al. 1999) in many applications, these biosensors have been widely used in virus and biological detection, and are considered as powerful tools for medical detection (Ribeiro et al. 2020; Pohanka 2018). Zhao et al. invented an ultra-sensitive and accurate electrochemical biosensor detection technology, which does not require nucleic acid amplification and reverse transcription, and can detect the RNA of SARS-CoV-2 through the super sandwich technology, and the detection rate is higher than that of RT-qPCR. The limit of detection (LOD) of clinical specimens was 200 copies/mL (Zhao et al. 2021). Qiu et al. invented a kind of dual-functional plasmonic photothermal biosensors that can detect the corresponding sequence of SARS-CoV-2 with an accurate detection limit of 0.22 pM. It also has high sensitivity to specific targets in multi-gene mixtures (Qiu et al. 2020). Due to the short life span of biosensors, their inability to be used for a long time and the need for multiple electronic devices such as bioreceptors, sensors and signal processing systems to work simultaneously, the further promotion of biosensors will be limited (Srinivasan and Tung 2015).

### Electrochemical biosensors

When utilizing an electrochemical biosensor (EC) for COVID-19, the helper protein of SARS-CoV-2 and the surface glycoprotein of E2 bind to the porphyrin of hemoglobin 1-β chain, thereby releasing heme. The biosensor’s receptor consists of a hemoglobin 1-beta chain, and if the specimen is positive, the SARS-CoV-2 protein binds to the hemoglobin on the sensor, producing an electrical signal. SARS-CoV-2 binds to hemoglobin on the sensor (Abid et al. 2021). EC offers several benefits, including low cost, simple operation, easy miniaturization and large-scale manufacturing. It can also be used as a bedside device at home or in a doctor’s office (Kumar et al. 2018).

### Optical biosensor

Optical biosensors are widely used analytical techniques that can detect biometric events through changes in optical signals. The detection technology of optical biosensors can be achieved by indirect or direct methods using absorption fluorescence, refraction, and surface plasmon resonance (SPR). Indirect optical biosensors rely on fluorophores to detect processes and amplify signals, producing high signals but suffering from nonspecific binding. On the other hand, surface-enhanced Raman scattering (SERS), is an ultra-sensitive molecular spectroscopy technique that is free from water interference, providing a clear advantage for biological sample identification. The direct approach of optical biosensors involves measuring the optical properties of the analyte and the sensing environment, such as SPR biosensors, by changing the refractive index of the interface between the subject and the sensor (Duan and Fan 2015). SPR is an optical detection method that uses the principle of prism reflection and can track biomolecular interactions in real-time in their native state (Neethirajan et al. 2017). To enhance the properties of ionic resonators for virus detection, nanomaterials are added to biosensors to expand the large biocompatible region with analytes, such as antibodies and DNA, and improve their specificity and sensitivity (Mokhtarzadeh et al. 2017). Additionally, viruses can be detected through indirect polymerization by modifying target molecules on the virus surface. The advantages of SPR include label-free and visual measurement with high reliability, sensitivity, and real-time performance. The design of SPR sensor devices is moving toward miniaturization, low cost, and user-friendliness (Neethirajan et al. 2017).

### Field-effect transistors

**Fig. 12:**
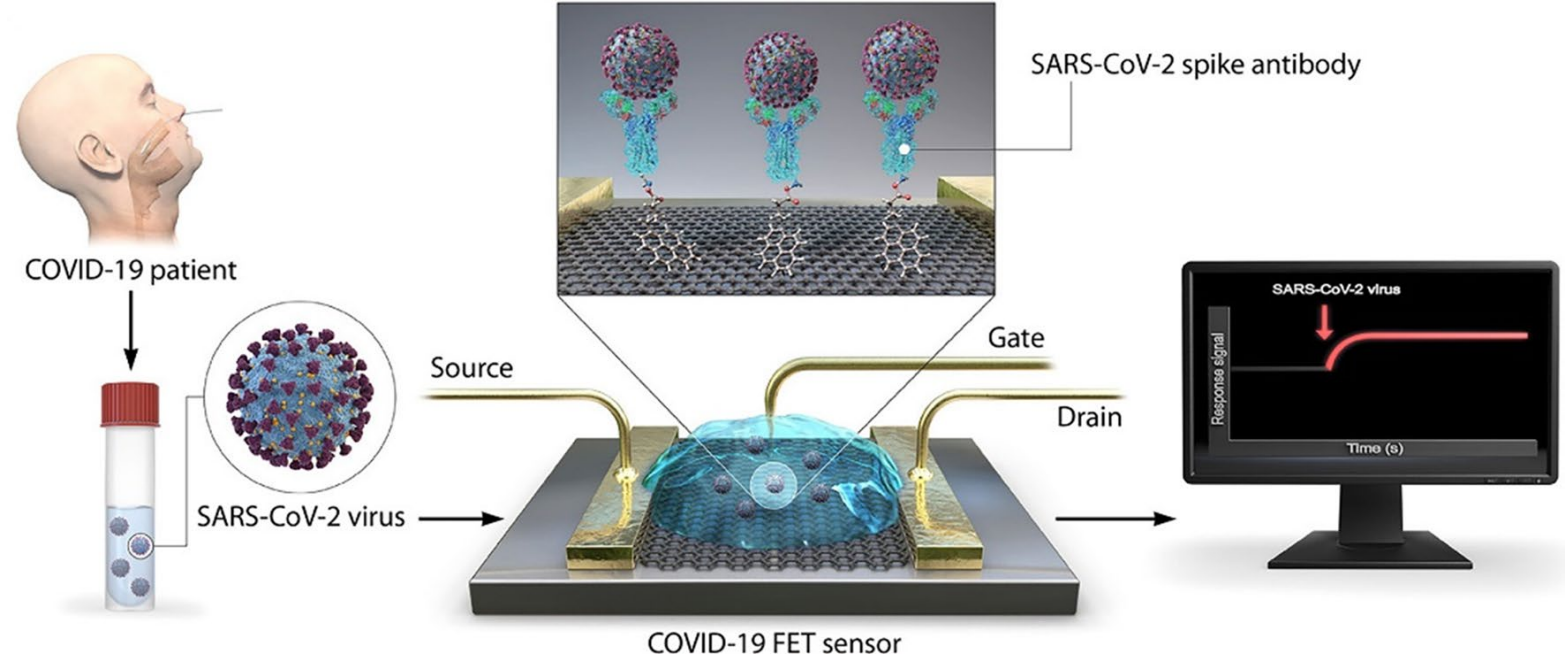
Point-of-care detection of SARS-CoV-2 antigen using a FET-based sensor. Upon binding of spike (S) proteins to the anti-S monoclonal antibodies immobilized on the graphene sheet via the PBASE linker, a change in the voltage-ampere diagram reveals the presence of the virus (Taleghani and Taghipour 2021)

Field-effect transistors (FET) are electronic biosensors that consist of trench-separated source and drain units, typically made entirely on silicon/silica substrates. A schematic of the detection changes performed by the graphene-based FET biosensor (Fig. 12) (Taleghani and Taghipour 2021). FETs offer several advantages, including simplicity of fabrication, low cost, and real-time detection (Seo et al. 2020).

Biosensors have emerged as effective tools for the early diagnosis, rapid and ultra-sensitive detection of SARS-CoV-2. Despite challenges and limitations in the biosensor design and application, the urgent global need for rapid detection of COVID-19 makes biosensors a unique option. Biosensor can reduce detection time, save costs, and minimize the transmission of COVID-19 compared to other infectious diseases.

### Methods to improve the speed of the detection

The RT-PCR is regarded as the gold standard for detecting SARS-CoV-2 infection, but it is expensive and time-consuming, taking 3–4 h to produce results. With the rapid spread of the pandemic, early and fast detection is crucial to prevent transmission and enable patients to receive prompt treatment to minimize complications (Misra et al. 2022). In addition, virus testing requires a high level of testing technology, is highly dependent on skilled personnel, and must be performed in well-equipped clinical laboratories. Therefore, there has been a shift towards developing effective diagnostic tools that are fast, sensitive, specific, cost-effective, and easy to use.

**Fig. 13:**
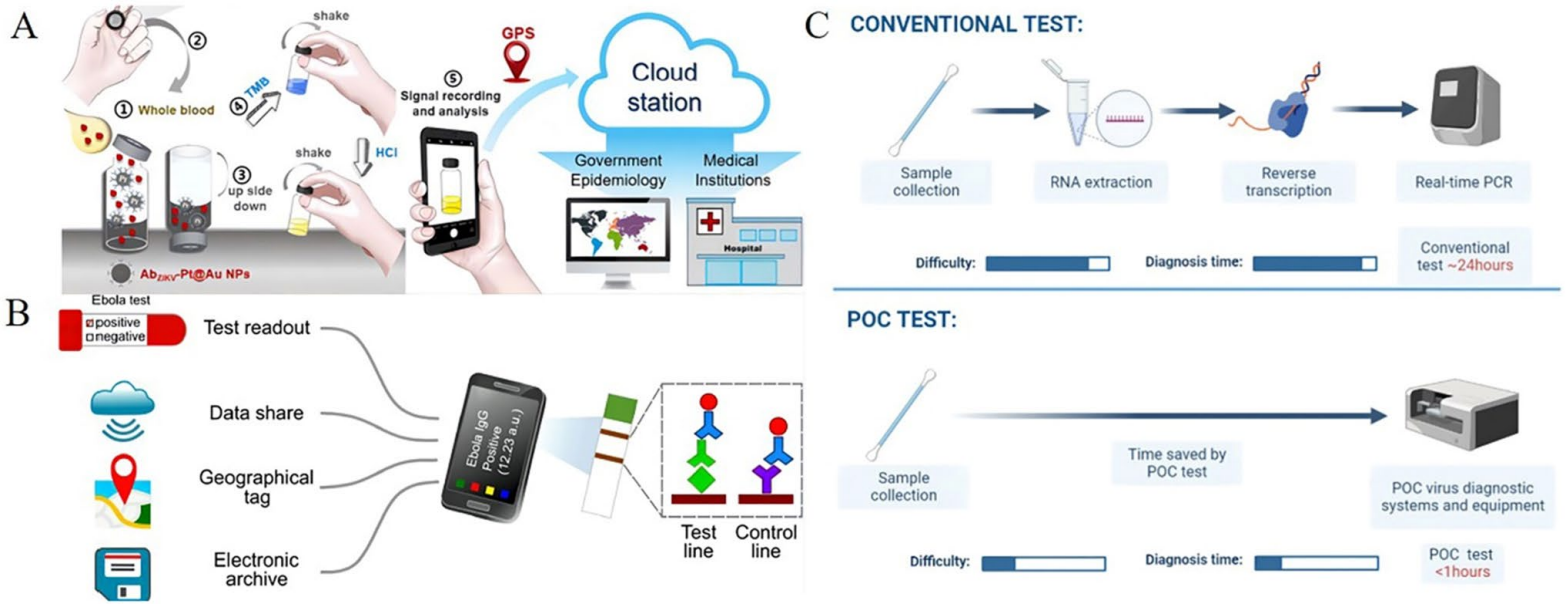
Colorimetric biosensors for virus detection on smartphones. **A** The ZIKV POC test’s instrument-free detection procedures (Hsu et al. 2020). **B** Ebola viral IgG detection with smartphones and colloidal gold LFIAS (Xiao et al. 2019). **C** Schematic illustration of conventional test and POC test (Saeed et al. 2017)

Advances in microfluidics, micro-electro-mechanical system (MEMS) technology, nanotechnology, 3D printing, and data analysis have led to the development of point-of-care testing (POCT), which is an ideal rapid detection method. Point-of-care (POC) biosensors based on chips and paper have been undergoing rapid innovation due to the COVID-19 pandemic (Rezaei et al. 2020). POC biosensors are ideal for detection due to their low-cost, require fewer reagent consumables, are easy to manufacture and operate, and have sample input and response capabilities (Choi 2020). In addition, with the popularity of smartphones, biosensor technology based on smartphones has the advantages of portability, speed, low cost, easy operation, and so on, which has a broad prospect (Fig. 13). POC biosensors allow for precise control and manipulation of liquids, require smaller sample sizes than traditional analytical methods, and can enhance the interaction between the analytical reagent and the target biomarker through efficient mixing of liquids, reducing detection time (Saeed et al. 2017). This not only benefits regional hospitals and centralized laboratories, but also facilitates testing outside the laboratory environment (Choi et al. 2017; Chinnadayyala et al. 2019).

**Fig. 14:**
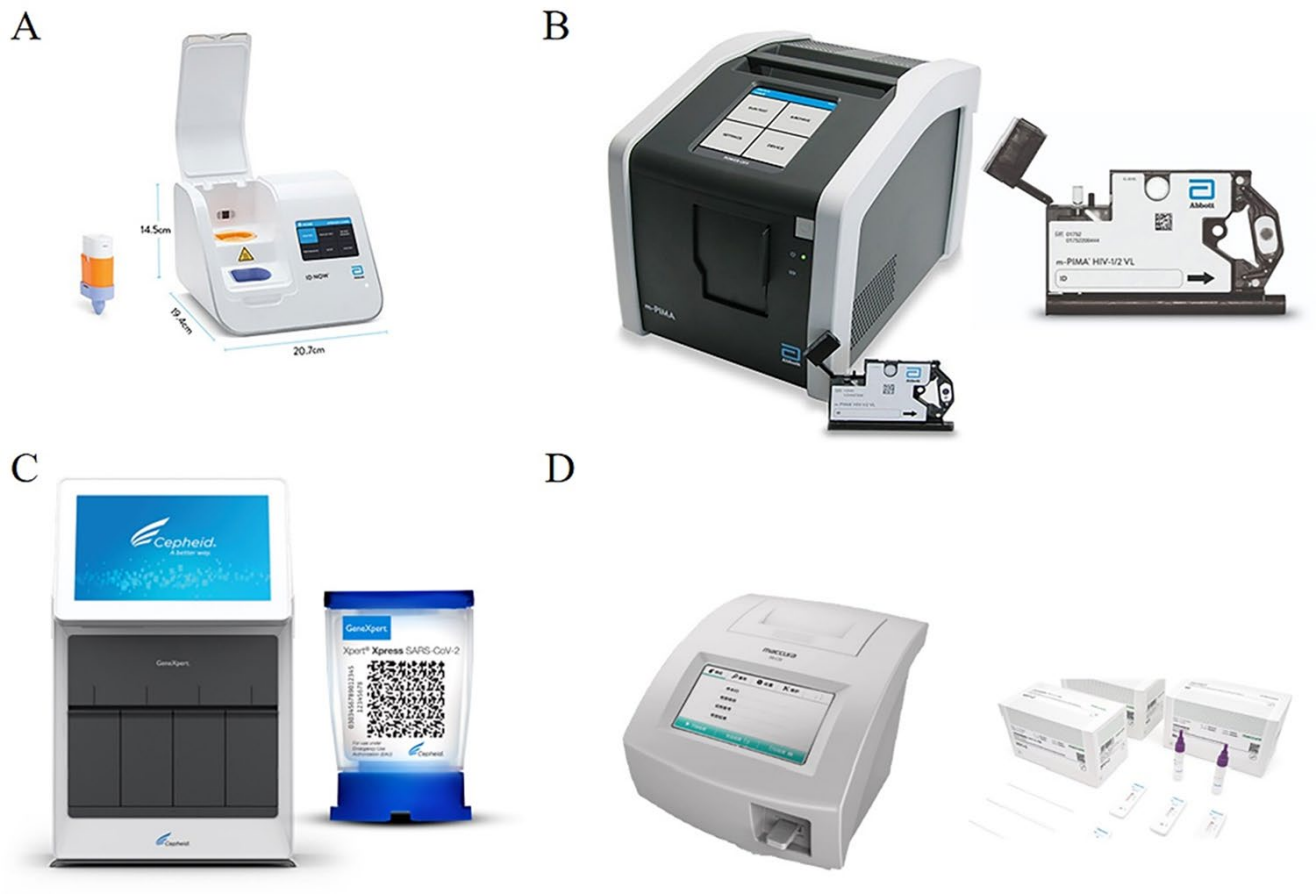
Systems and tools for POC viral diagnosis are available today. **A** ID NOW influenza A & B 2 system (https://www.globalpointofcare.abbott/en/product-details/id-now-influenza-ab-2.html). **B** The m-PIMA analyzer and m-the PIMA HIV-1/2 test cartridge (https://www.globalpointofcare.abbott/en/product-details/m-pima-analyser.html). **C** The GeneXpert System and Xpert Xpress SARS-CoV-2^®^ (https://www.cepheid.com/en_US/systems/GeneXpert-Family-of-Systems/GeneXpert-System). **D** R01 fluorescence immunoassay analyzer and Rapid test strip (https://www.maccura.com/en/product/uwQA2OQdXAE-.html)

There are three main POCT methods for detecting SARS-CoV-2: nucleic acid detection, serological immunoassay, and biosensor detection. The fluid from the nasopharynx or oropharynx is typically collected for nucleic acid testing, followed by the isolation of the virus. These products can filter and purify nucleic acids, scale down PCR testing, centralize management, reduce reagents and waste, energy consumption, and improve test efficiency (Su et al. 2015). The LAMP-based detection method has gained popularity in SARS-CoV-2 detection of POCT due to its fast detection speed, simple heating requirements, and other benefits. LAMP-based POC equipment combines paper-based POC diagnostic devices with LAMP analysis technology to achieve rapid and convenient tests for SARS-CoV-2 (Yang et al. 2020). Moreover, chromatographic strip detection results can be directly observed by the naked eye, and CRISPR-based results can be identified by tablet readers, lateral flow visualization, or fluorescent colors, making this method suitable for POCT (Huang et al. 2020a, b). Immunoassay is divided into lateral flow immunoassay (LFA), ELISA, and chemiluminescence. The LFA method has the advantage of fast detection speed and low cost. ELISA and chemiluminescence are more accurate and dependable than LFA (Ko et al. 2020; Coste et al. 2021). Therefore, colloidal gold/immunofluorescence chromatography is a promising, rapid, and portable POC immunodetection platform (Wen et al. 2020). Currently, some POC virus diagnosis systems and equipment are available on the market (Fig. 14).

However, most POC devices used to detect COVID-19 disease are authorized only in emergencies, and thus, caution must be exercised when diagnosing other diseases with such devices. Despite significant advances, biosensors still have a lot of room to improve in terms of sample pretreatment and preparation costs, false negatives and positives, and selective limitations. The apparent gap between scientific theoretical research and actual products may be due to low stability, high cost, and patent restrictions. Additionally, integrating different detection strategies and platforms to develop new mixing equipment may improve performance.

## Methods to improve the quality of the detection

Nucleic acid detection is the primary technology for early diagnosis of novel coronavirus infection and a crucial tool in the battle against COVID-19. Rapid and accurate detection of novel coronavirus infections is essential to prevent the spread of the epidemic. The timely identification of infected individuals helps to isolate, diagnose, and treat them, reducing the risk of transmission. False or missed tests will result in false negative carriers not being effectively isolated and treated, thus becoming new sources of infection. Consequently, the quantitative accuracy of nucleic acid test results has become crucial concern for society.

### Quality control before analysis

#### Sample collection

SARS-CoV-2 is mainly found in the lower respiratory tract. Theoretically, the lower respiratory tract or bronchoalveolar fluid should be tested. However, in clinical practice, the collection of samples such as nasopharyngeal swabs, saliva, oropharyngeal swabs, and nasal swabs are commonly used, with nasopharyngeal swabs being the most important criteria for diagnosing SARS-CoV-2. To improve detection efficiency, multiple sampling methods (such as simultaneous oropharyngeal and nasopharyngeal swabs) are recommended for the same case. Standardized sample collection procedures are important to avoid excessive force during sampling that can cause coughing or sneezing, which can lead to high local concentrations of droplets and aerosols, resulting in cross-contamination between samples. At the same time, avoid eating and drinking water when collecting samples (Todsen et al. 2021; Lee et al. 2021a, b; Zou et al. 2020).

#### Sample storage and transportation

Standard operating procedures for specimen collection should be formulated in daily work, and systematic training and assessment of specimen collection and inspection personnel should be strengthened, including personal protective operation, sample types and collection methods, sample collection operation procedures and precautions, etc., ensure that the specimen is not contaminated during collection, transportation, and storage.

To improve the sensitivity of the COVID-19 test, a nucleic acid stabilized mixture (NSLB) can be added to the virus lysis buffer during sample collection (Erster et al. 2021). After the sample is collected, the specimen cap should be tightly sealed with an appropriately sized disposable sealing bag and placed in a temporary storage box for low-temperature storage (Yilmaz Gulec et al. 2021). The sample should be transported for testing as soon as possible, and the outer surface of the sealed bag should be disinfected with 75% alcohol or a 2000 mg/L chlorine-containing disinfectant to prevent contamination during transportation. It is recommended to use special biosafety class A containers that are resistant to high and low temperatures, pressure, and have good sealing for specimen transportation (Karthik et al. 2020). After placing the sample in the transfer box, the external surface of the box should be disinfected with disinfectant (Table 6) to prevent cross-contamination caused by violent shaking during dumping or transfer.

**Table 6:** Effective disinfectant for SARS coronavirus

Type of disinfectant	Concentration	Contact time	References
Povidone iodine	0.23–7.5%	15 s	Wu et al. (2020a, b); Mariano et al. (2020)
Ethanol	78–95%	30 s	Corman et al. (2020); Chan et al. (2020)
2- Propanol	70–100%	30 s	Alagarasu et al. (2020)
Sodium hypochlorite	0.21%	1 min	Chu et al. (2020); Tombuloglu et al. (2021)
Hydrogen peroxide	0.5%	1 min	Wang et al. (2020a, b, c); Kim et al. (2022)
Formaldehyde	0.7–1%	2 min	Alagarasu et al. (2020)

#### Detection environment

Maintaining a standard laboratory is crucial in ensuring the accuracy of the test, as the virus can exist in aerosol form in the air and on object surfaces for extended periods. Laboratory contamination can lead to false positive results. To prevent this, the PCR laboratory should strictly implement independent zones for reagent preparation, nucleic acid extraction, amplification, and other processes, with a one-way flow of personnel and logistics. It is suggested to place unadded samples in biosafety cabinets, refrigerators, and counter boards during nucleic acid testing and amplify them simultaneously with samples to monitor any possible laboratory contamination (Y.Q. et al.2020).

### Quality control during detection

To ensure accurate results in nucleic acid extraction, reaction mixture preparation, and amplification, it is important to maintain unidirectional flow of samples and prevent cross-contamination between specimens or between specimens and positive controls. After testing each batch of samples, the nucleic acid extractor should be disinfected with ultraviolet lamps. Additionally, using different pipettes and filtered tips when preparing samples and reaction mixtures can help prevent contamination (Gupta 2020).

Various factors can affect PCR experiments, including the quality and content of viral nucleic acid, which can reduce the stability of reagents and result in unstable test results. To address this, it is recommended to include one weakly positive quality control item and three negative quality control items in each batch of samples, with the negative items placed randomly in different locations. In addition, the improper operation is also an important cause of cross-contamination, resulting in false positive results.

## Conclusions

Among the current novel coronavirus detection methods, RT-PCR is still the “gold standard” for novel coronavirus detection. However, limited resources such as PCR detection kits, coupled with long turnaround times, often lead to shortages during pandemics. High-volume outbreaks can strain laboratories and increase the risk of sample contamination. Additionally, RT-PCR presents challenges in remote and underdeveloped areas. Therefore, it is essential to develop COVID-19 detection technologies suitable for different scenarios. POCT based on CRISPR and RT-LAMP can achieve quick, low-cost, and portable detection of the novel coronavirus, making them suitable for use in airports, border ports, and remote regions, and possibly even for home testing. However, low stability, high costs, and restricted patents create a significant gap between scientific theoretical research and actual product development. Therefore, multiple testing methods should complement each other to cover various stages of COVID-19 development, such as screening positive patients, confirming infection, and treating the disease. So far, the epidemic has lasted more than three years, but the WHO has not declared the global epidemic over, and the daily death toll continues to rise. Early, fast and accurate testing of all infected people, even asymptomatic carriers of COVID-19, is critical. Therefore, there is still a need to continue to refine and develop more effective assays. Until effective treatment methods are developed, complete lockdown, mask-wearing, and vaccination remain the most effective measures to prevent novel coronavirus spread and pandemic.

## Data Availability

The data underlying this article are available in the article.
